# Deliberate Practice and Proposed Limits on the Effects of Practice on the Acquisition of Expert Performance: Why the Original Definition Matters and Recommendations for Future Research

**DOI:** 10.3389/fpsyg.2019.02396

**Published:** 2019-10-25

**Authors:** K. Anders Ericsson, Kyle W. Harwell

**Affiliations:** Department of Psychology, Florida State University, Tallahassee, FL, United States

**Keywords:** deliberate practice, expert performance, mental representation, practice effects, heritability

## Abstract

Over 25 years ago Ericsson et al. (1993) published the results of their search for the most effective forms of training in music, a domain where knowledge of effective training has been accumulated over centuries. At music academies master teachers provide students individualized instruction and help them identify goals and methods for their practice sessions between meetings – this form of solitary practice was named *deliberate practice*, and its accumulated duration during development was found to distinguish groups with differing levels of attained music performance. In an influential meta-analysis Macnamara et al. (2014) identified studies that had collected estimates of practice accumulated during development and attained performance and reported that individual differences in deliberate practice accounted for only 14% of variance in performance. Their definition of “deliberate practice” differs significantly from the original definition of deliberate practice and will henceforth be referred to as *structured practice*. We explicate three criteria for reproducible performance and purposeful/deliberate practice and exclude all effect sizes considered by Macnamara et al. (2014) that were based on data not meeting these criteria. A reanalysis of the remaining effects estimated that accumulated duration of practice explained considerably more variance in performance (29 and 61% after attenuation correction). We also address the argument that the limited amount of variance explained by the duration of practice necessarily implies an important role of genetic factors, and we report that genetic effects have so far accounted for remarkably small amounts of variance – with exception of genetic influences of height and body size. The paper concludes with recommendations for how future research on purposeful and deliberate practice can go beyond recording only the duration of practice to measuring the quality of practice involving concentration, analysis, and problem solving to identify conditions for the most effective forms of training.

## Introduction

Since the publication of Ericsson, Krampe, and Tesch-Römer’s article on “The Role of Deliberate Practice in the Acquisition of Expert Performance” in 1993, the concept of deliberate practice has received a lot of attention. In the fall of 2019, over 25 year later, Google Scholar reported over 10,000 citations of that article and over 35,000 articles containing the word combination “deliberate practice” from 1993 to date, compared to fewer than 500 cases before 1993. It is important to note that Ericsson et al. (1993) defined the term *deliberate practice* as the individualized solitary practice in classical instrumental music as directed by a qualified teacher. This type of practice requires that several different criteria are met. Some early investigators noticed that the conditions for deliberate practice were rarely met in sports (Starkes et al., 1996). More recently Baker et al. (2005, p. 65) argued that deliberate practice is “predicated on the concept that it is not simply training of any type, but the engagement in specific forms of practice, that is necessary for the attainment of expertise”. Deliberate practice was presented (Ericsson et al., 1993) as the result of a search for evidence on optimal learning and improvement of performance. This research was an effort to explore if one could find examples in everyday life corresponding to the surprisingly large improvements in memory performance (over 1,000%) demonstrated by a college student after engaging in hundreds of hours of extended practice (Ericsson et al., 1980).

The domain of music has historically utilized individualized training of full-time students by teachers and has accumulated knowledge about effective training for several centuries. At an international music academy, the best violinists were compared to less accomplished expert violinists and were found to have engaged in more solitary practice during their musical development (Ericsson et al., 1993). Subsequent research documented that the increased amount of certain types of practice was correlated with higher levels of attained performance in a wide range of domains (Ericsson, 1996, 2003, 2007; Ericsson and Lehmann, 1996; Ericsson et al., 2018). These findings stimulated a number of journal editors to assign special issues that focus on discussions on the role of nature and nurture in the development of expertise in journals such as *International Journal of Sport Psychology* (Baker and Davids, 2007), *High Ability Studies* (Stoeger, 2007), and *Intelligence* (Detterman, 2014).

Over a decade after the original publication of the paper proposing deliberate practice, Gladwell (2008) published his very popular book *Outliers*, and he dedicated a whole chapter to the topic of the “10,000 h rule” and cited our paper (Ericsson et al., 1993) as the primary empirical evidence for the rule. In that chapter Gladwell (2008) proposed that a minimum of hours of practice was necessary and that this number was “the magic number for true expertise: ten thousand hours.” Although our research showed that an extended period of training and practice was required for attaining international-level performance, there was no evidence for a magical number. In fact, to win international piano competitions the first author estimated that around 25,000 h would be more accurate (Ericsson, 2013). Even more significantly, Gladwell (2008) never mentioned the term “deliberate practice” in his book and only referred to practice in general. His discussed examples of individuals surpassing the 10,000 h boundary to world-class success explicitly included many types of practice activities that were violating the criteria for deliberate practice, such as public performances and work. As is often the case when ideas are popularized, they become simplified and lose their original meaning. The 10,000 h rule was interpreted as saying that unless one has engaged in an activity for 10,000 h one will not have been able to reach excellence and mastery. The more popular interpretation says “‘10,000 h’ succeeds as a meme because it tells people what they want to believe, that with enough practice, anyone can covet the skills of genius. It’s not so much that people want to become world-class musicians or top physicists, but rather that they have the potential to become those things if they want to, by practicing enough” (Hacker news, 2017). The essence of the popular belief is that the critical factor determining one’s attained performance is how long one has been practicing, which could be measured by the number of estimated hours that a given individual has practiced.

It is possible to assess the validity of this belief by conducting a meta-analysis of the correlation between the accumulated amount of practice and attained performance in a wide range of domains. Macnamara et al. (2014) conducted the first meta-analysis and they identified over 9,000 studies that matched keywords, such as “practice,” “deliberate practice,” and many other related terms. They also required that “the study report referred to at least one publication on deliberate practice by Ericsson and his colleagues” (p. 1610), and that the study report provided information on an accumulated amount of practice and a measure or index of performance. Studies meeting these criteria contributed the data for their meta-analysis. Macnamara et al. (2014) claimed their analysis would evaluate Ericsson et al. (1993) “influential deliberate-practice view of expert performance. This view holds that expert performance *largely reflects accumulated amount of deliberate practice*” (Macnamara et al., 2014, p. 1608, italics added). Out of the many studies identified they selected 88 studies which had measured “accumulated amount of one or more activities *interpretable* as deliberate practice” (p. 1611, italics added). They did not use the definition proposed in the original paper (Ericsson et al., 1993), but selected a more general description from the paper (the differences between definitions will be discussed in more detail later in this paper). They interpreted the definition to be as follows: “*deliberate practice*, which was defined as engagement in structured activities created specifically to improve performance in a domain” (Macnamara et al., 2014, p. 1608). Their meta-analysis concluded: “We found that deliberate practice explained 26% of the variance in performance for games, 21% for music, 18% for sports, 4% for education, and less than 1% for professions. We conclude that deliberate practice is important, but not as important as has been argued” (Macnamara et al., 2014, p. 1608). They claimed that their results estimated the relation between attained reproducibly superior performance and the accumulated amount of deliberate practice, but we disagree and will show that their definition of “deliberate practice” included a much broader set of activities, such as many types of domain-specific experiences and competitive events. Drawing on Macnamara et al. (2014, 2016) published claims about deliberate practice, many researchers cited the results of this meta-analysis to show the limits of any type of practice in influencing performance. For example, some scientists studying sport cited those estimates in support for their claim that the remaining factors are “substantially heritable in nature” (Georgiades et al., 2017, p. 62), and thus, by inference, elite sport performance is primarily determined by individual differences in genes. In a recent review article Moreau et al. (2018) cited a meta-analysis of studies analyzed by Macnamara et al. (2014) but with a restriction to only studies of sports performance (Macnamara et al., 2016). This meta-analysis was cited to show that deliberate practice could not explain any statistically significant amount of the variance of individual differences “in performance among elite-level performers” (Moreau et al., 2018, p. 333). Similarly, Thomas and Lawrence (2018) reviewed expert performance across different professional domains and claimed that “deliberate practice fails to account for large proportions of variance in expertise” (p. 171).

These claims about the limitations of deliberate practice to influence attained levels of performance are based on Macnamara et al. (2014) meta-analysis. In this paper we will show that the definition adopted in the two meta-analyses (Macnamara et al., 2014, 2016) led to the inclusion of data on performance and practice that did not meet the criteria for deliberate practice and reproducible performance originally proposed by Ericsson et al. (1993). In the next section we will describe how the original definition of deliberate practice was generated, and in the immediately following section we will describe some problems that investigators had in identifying practice meeting all the criteria for deliberate practice in domains of expertise other than music and how this led to the need to distinguish and identify criteria for several different types of practice.

In the main body of this paper we will examine how our conceptions of deliberate practice and reproducible performance has implications for which studies in Macnamara et al. (2014, 2016) meta-analysis reflect deliberate and purposeful practice and therefore provide valid information about the relation between accumulated deliberate and purposeful practice and attained performance. We will sequentially apply three criteria to allow us to identify the small subset that can be agreed upon as representing valid information to be aggregated to estimate the relation between the accumulated estimated duration of deliberate and purposeful practice and attained reproducible performance. We will then calculate how this estimate can be corrected for attenuation for reliability and discuss issues related to restriction of range when the general hypothesis considered is “how much variance can practice account for when analyzing individual differences in performance.” In addition, we discuss several issues regarding the strength of the relation between accumulated amount of deliberate and purposeful practice and attained reproducible performance, as well as why such aggregated estimates will never provide accurate estimates of the upper-bound for how much accumulated durations of high-quality practice can account for improvements of performance. The concluding sections propose how research on genetics and on detailed training histories can be combined to assess the relative role of these respective factors and their possible interactions in predicting the level of performance attained in the particular domain of expertise. In our final section, recommendations for future research on the development and acquisition of expert performance will be presented.

## The Original Definition of Deliberate Practice

The original stimulus for the work on deliberate practice came from the goal of finding effective training for attaining expert levels of performance in professional domains outside the laboratory. In the research collaboration with Bill Chase (Ericsson et al., 1980; Chase and Ericsson, 1981, 1982), college students with average performance on ability tests were shown to be able to dramatically increase their memory performance by engaging in several hundred hour-long laboratory sessions of practice distributed over more than a year. Consequently, Ralf Krampe, Clemens Tesch- Römer and the first author started our research by searching for “conditions for optimal learning and improvement of performance” (Ericsson et al., 1993, p. 367). This paper reviewed a century of laboratory studies of learning showed that performance was increased when participants “attend to the task and exert effort to improve their performance…. The subjects should receive immediate informative feedback and knowledge of results of their performance. The subjects should repeatedly perform the same or similar tasks” (p. 367). The review reported on a search for such activities with explicit goals and immediate feedback and identified a domain of expertise with centuries of successful production of expert performers, namely the training of instrumental musicians. Consequently, this paper examined the daily activities of music students attending an internationally renowned music academy. It was assumed that the select students admitted to the music academy were highly motivated to improve their performance to prepare for their professional careers and thus able and willing to “attend to the task and exert effort to improve their performance” (p. 367). There were only two types of activities that focused on explicit goals of improving aspects of individual performance with established practice activities that offered immediate feedback and opportunities for repetition after reflection. The first type of activity involved individual students’ lessons with their teacher, but those lessons are typically restricted to around only 1 h per week. It was observed, however, that the teacher influenced and guided practice activities beyond the lesson and “the teacher designs practice activities that the individual can engage between meetings with the teacher. We call these practice activities *deliberate practice*” (Ericsson et al., 1993, p. 368). More specifically, “[T]to assure effective learning, subjects ideally should be given explicit instructions about the best method and be supervised by a teacher to allow individualized diagnosis of errors, informative feedback, and remedial part training. The instructor has to organize the sequence of appropriate training tasks and monitor improvement to decide when transitions to more complex and challenging tasks are appropriate” (Ericsson et al., 1993, p. 367). This paper pointed out that the activity of deliberate-practice is a particularly interesting locus of individual differences in the amount of practice because the music students can control when and for how long they engage in this type of practice. Consequently, this paper hypothesized that students who consistently engaged in more hours of this type of “practice alone” per week would be predicted to improve their music performance significantly more.

**FIGURE 1:**
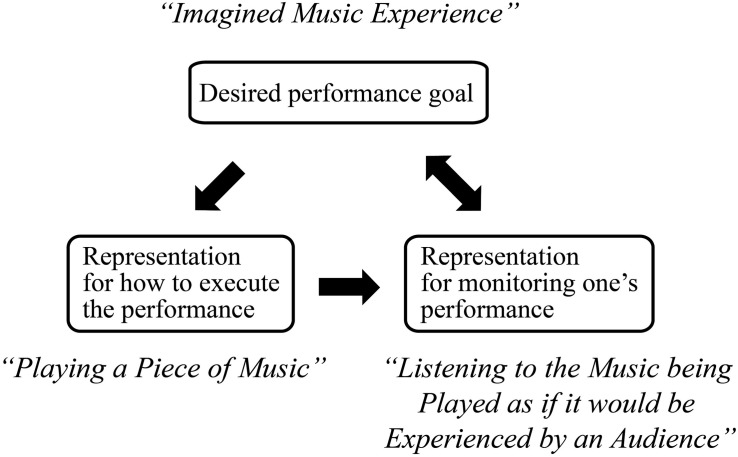
Three types of systems of mental representations that mediate expert music performance and its continued improvement during deliberate practice. Adapted from Figure 6 in Ericsson (1998).

Deliberate practice differs qualitatively from most other forms of practice. The first criterion is that the practice involves individualized training of a trainee by a well-qualified teacher. This teacher can assess which aspects a particular trainee would be able to improve during the time until the next meeting and is able to recommend practice techniques with established effectiveness. The second criterion is that the teacher must be able to communicate the goal to be achieved by the trainee and that the trainee can internally represent this goal during practice. It is challenging for the trainees to be able to mentally represent a goal for a level of performance that the trainee is initially unable to attain. For example, to attain successful mastery of a music piece (see Figure 1) the trainees need to be able to mentally represent the desired sound of the piece of music (top box in Figure 1) in order to be able to generate controlled attempts with their instruments that gradually approach this goal (left lower box). The third criterion is that the teacher can describe a practice activity to attain the identified goal for performance and that this activity allows the trainee to get immediate feedback on a given attempt. For example, a musician would be able to listen to the sound of their produced attempt (right lower box) and be able to notice differences between the sound of their desired goal and of their current attempt and then make the differences targets for generating a new and better attempt after opportunities for reflection and problem solving. The fourth criterion is that the trainee is able to make repeated revised attempts that gradually approach the desired goal performance.

There is an additional feedback cycle that occurs when trainees return to their teacher for their lessons and demonstrate the performance that they had worked on throughout the week. The students will get feedback on how well they attained the assigned goals from the previous meeting and the teacher can help the trainees to refine their mental representations so they can reliably notice differences that have not yet been successfully addressed. Once current practice goals have been attained, the music teacher identifies new goals and associated valid practice activities to allow a particular student to keep improving their performance with additional deliberate practice. The deliberate practice framework argues that expert performers continue to strive to attain more refined mental representations, which provide increased ability to control performance. This is in direct contrast to traditional theories of acquisition of everyday skills (Fitts and Posner, 1967), where individuals try to automate their behavior within weeks or months to minimize effort. In support of the expert performance account, expert performers are able to verbalize their thoughts during planning and evaluation of their performance when they are asked to “think aloud” (Ericsson, 2018a) and are able to recall much more relevant information encountered during a brief exposure to a challenging situation than their less accomplished peers (Ericsson, 2018b).

## Challenges in Efforts to Find Deliberate Practice in Domains Other Than Music

As mentioned earlier, researchers in different domains reported difficulties finding practice activities that exactly matched the individualized one-on-one training in music. For example, Starkes et al. (1996) searched for practice activities in different sports that were rated as highly related to improvement, requiring a high level of concentration and would not be enjoyable, but were not able to find activities that met all three criteria. Other pioneering investigators, such as Charness et al. (1996) focused on a single particular domain, namely chess, and searched for a domain-specific practice activities such as “serious analysis of positions alone (chess books, magazines, databases, postal chess, etc.)” (p. 75) that they referred to as “serious practice alone” or deliberate practice alternatively. They also gathered information on the amount of time that the chess players had spent with a coach and found that coached players attained higher chess ratings, but also that coached players spent more time on serious practice alone so there was not an independent benefit of amount of coaching in that sample. However, Ericsson and Charness (1994) had stated clearly that “self-directed study has most of the characteristics of deliberate practice, but it is probably not as effective as individualized study guided by a skilled teacher” (p. 739). Other researchers started exploring less well-defined domains of expertise. Dunn and Shriner (1999) interviewed teachers to identify “deliberate practice as those activities which are highly relevant to improving performance and require significant personal effort to initiate and maintain” (p. 632) within the domain of teaching. They had teachers rate different types of practice activities and identified practice activities that had high ratings of relevance for self-improvement as teachers, perceived effort and frequency of occurrence, such as “preparing materials needed for instructional activities” (p. 636). In a second study they had expert teachers, defined as someone with over 10 years of experience, keep a diary of their different activities during a week. This study is an interesting effort to identify activities, referred to as deliberate practice, that some expert teachers might engage in to keep improving their teaching performance throughout their career. However, there was no objective measurement of teaching performance, nor any identification of specific goals for improving aspects of performance along with effective practice tasks and no supervision of training by a skilled teacher. In several domains of expertise, researchers have become interested in the idea of identifying practice activities that experts would have engaged in to reach a superior level of objective performance. At the same time, it is essential to distinguish the search for such activities from the original definition of deliberate practice (Ericsson et al., 1993), which referred to individualized training designed by a teacher in a domain with a well-developed knowledge about effective methods for improving aspects of performance.

When Ericsson et al. (1993) defined deliberate practice, they created a problem by only introducing a single new concept. There are a range of practice activities that do not meet all the criteria for deliberate practice but are still associated with performance gains. More recently Ericsson and Pool (2016) addressed this conceptual confusion and proposed the term *purposeful practice* for individualized practice activities which the trainee engages in to improve their performance but without the benefit of a teacher with extensive knowledge of effective methods for practice. This type of practice is well illustrated by the serious practice alone in chess (Charness et al., 1996), in SCRABBLE (Moxley et al., 2019), in darts (Duffy et al., 2004), in bowling (Harris, 2008) and many individual sports, such as running (Young and Salmela, 2010). In addition, Ericsson and Pool (2016) proposed the term *naïve practice*, for practice involving merely engaging in domain-relevant activities, such as playing games with friends and others in tennis, golf, and soccer. In the case of people working in various professions, naïve practice would involve simply executing the job in response to demands evolving normally by external factors.

## Assessing the Modifiability of Expert Performance in Response to Deliberate Practice

It is challenging to attempt to measure the maximal degree to which practice can influence the level of attained performance, but it is possible to identify several necessary steps. The first step would involve describing and clearly defining the type of practice that shall be examined. This is of crucial importance for studies of deliberate practice, which was defined to be a very different type of practice compared to the typical engagement in activities in the domain. The second step involves explicating how one can describe and measure individual differences in the amount and quality of practice activities accumulated during an individual’s prior development in the domain. A related issue concerns the possibility of creating indices that would quantify some of these differences that would allow one to relate individual differences in aspects of accumulated practice to attained performance. The third step involves the assessment of the relations between these indices of practice and the level of attained performance. In a subsequent section we will discuss how the relations between accumulated practice and attained performance, as well as other types of evidence, provide information about the limits of practice to improve performance.

### Differences in the Two Definitions of Deliberate Practice by Ericsson et al. (1993) and Macnamara et al. (2014, 2016)

Earlier we described how different researchers attempted to identify deliberate practice in domains other than music. The most general idea, which was stimulated by our work on deliberate practice, led to the search for activities that motivated individuals engage in with the explicit goal of attempting to improve their reproducibly superior performance in some domain of expertise. Consistent with their search for deliberate practice, Macnamara et al. (2014, p. 1608, italics added) introduced their definition of “deliberate practice, which Ericsson et al. *defined* as engagement in structured activities created specifically to improve performance in a domain.” Hambrick et al. (2014) similarly included the emphasis on activities that have been specially designed to improve the current level of performance. There is no disagreement that the goal of improving performance is one characteristic of deliberate practice, and Ericsson et al. (1993) even wrote that “deliberate practice is a highly structured activity, the explicit goal of which is to improve performance” (p. 368). This sentence was, however, not a definition of deliberate practice any more than the true statement that “a dog is an animal” would imply the inference that “all animals are dogs.” To avoid confusion between our original definition of deliberate practice and the definition of deliberate practice presented by Macnamara, Hambrick, and their colleagues, we will refer to their definition as *structured practice*, which is consistent with a terminology proposed by Hüttermann et al. (2014).

Macnamara et al. (2014) definition of structured practice is very broad and would include a number of practice activities designed by teachers, students, groups and individuals for the purpose of improving. There is much less of a problem for Hambrick et al. (2014) because they restricted their meta-analysis to studies from only two domains of expertise, namely chess and music, where a couple of the pioneering studies proposed well-documented training activities. The definition of structured practice has direct consequences for the inclusion of data sets in Macnamara et al. (2014) meta-analysis. For example, some of the included studies examined nurse education (Snelling et al., 2010) and measured the number of hours spent at lectures and seminars as a measure of accumulated practice. More generally, many other included studies used self-reports of hours of studying as the only measure of hours of accumulated structured practice. These studies met Macnamara et al. (2014) criteria for inclusion because they all cited the same paper, where the first author was a co-author. In this paper, Plant et al. (2005) proposed that studying was not deliberate practice but stated in the title that there were “Implications of Deliberate Practice for Academic Performance” (p. 96). The whole paper was dedicated to a proposal “that distinctions between deliberate practice and other types of practice can be applied to studying and that this distinction can, at least in part, explain why measures combining all types of study activities in the school system are not valid predictors of grades” (p. 99). Further evidence that including these papers on education and studying was not appropriate for an evaluation of deliberate practice is apparent by finding that nearly all of these studies only cited the Plant et al. (2005) study and did not even mention the term deliberate practice in the text of their articles. More generally, the practice activities involved in students’ study of material in a single course cannot be isolated from their prior learning for over a decade in the school system, and additionally the structure of these activities are not sufficiently well understood to allow us to categorize this type of practice in a meaningful manner (Plant et al., 2005). Their results will not be considered further in this paper. Other studies of team sports collected number of hours engaged in organized activities for teams. For example, Helsen et al. (1998) studied soccer players’ team practice that focused on games, tactics, technical skills and individual activities, such as running and weight training. When teams do training in groups, it is often not possible to individualize the training for each player. It is important to point out that organized team training may be quite effective in improving performance, but it does not meet all the criteria for deliberate practice.

The estimates used by Macnamara et al. (2014) consistently aggregated qualitatively different types of activities. For example, Baker et al. (2003) reported estimated amount of time spent in different activities for state and international-level athletes. Macnamara et al. aggregated the differences across all activities even though for some of the included activities, such as number of hours watching their sport on television, the state-level athletes spent nearly twice as many hours than the international level athletes. In other activities, such as hours of individual instruction with a coach, the international-level athletes report spending eight times as many hours as the state-level athletes. Similarly, Macnamara et al. (2014) aggregated correlations of hours of practicing alone and with others into a single estimate for the players of bowling in the study by Harris (2008).

Rather than simply criticizing the inclusion of effect sizes from these studies in these meta-analyses, we will propose how the studies could be re-analyzed in a manner that shows how these effect sizes, or at least some of the effect sizes, can provide information relevant to our research on aspects of deliberate practice. We will organize the effects with practice activities according to the three types of practice distinguished by Ericsson and Pool (2016), namely deliberate, purposeful and naïve practice as well as structured practice as proposed by Hüttermann et al. (2014).

#### Deliberate Practice

When we examined any practice activities reported in all the studies included in the meta-analyses, we found that very few of them met all the criteria for our definition of deliberate practice. Only a small minority described teachers/coaches assessing the individual performance of trainees and then recommending particular practice activities with immediate feedback.

#### Purposeful Practice

We found a considerably larger number of practice activities where trainees were engaging in solitary practice with the goal of improving particular aspects of performance without the regular access to individualized evaluation and guidance by a particular coach or teacher. This type of solitary practice is hardly ever completely independent of teachers and their knowledge about effective training. It is likely that these individuals had occasional meetings with a coach, discussions with more advanced athletes within the same sporting event, or reading books describing appropriate practice activities. Although individuals practice by themselves, some of them will know about practice activities with immediate feedback on their performance, such as interval training and training with weights, and thus engage in reasonably effective practice even without having a coach monitor and guide the detailed goals for their practice. To deal with this problem, we will be conservative and classify solitary practice activities as purposeful practice when these activities are not conducted with regular individual meetings and guidance from a particular teacher or coach. In addition, we excluded estimates of practice where it was not possible to determine how much of the time was spent in practice activities meeting the criteria for purposeful practice.

#### Structured Practice

This type of practice activity is best exemplified by the structured practice activity guided by coaches of teams or teachers of groups of students. That is, trainees engage in group activities designed by a coach or teacher, and these activities are not individualized and tailored to their current level of skill and opportunities to improve specific aspects of their current performance. Many of the practice estimates extracted from the included sports studies would be most appropriately classified as involving structured practice.

#### Naïve Practice

In the description of the characteristics of deliberate practice Ericsson et al. (1993) explicitly contrasted it to work and play activities, which both are motivated by other factors than the goal of improving a particular aspect of performance. The primary problem with many estimates of hours of engagement in practice activities is that the included practice activities are so broad that they most certainly include a considerable proportion of naïve practice. For example, organized practice for teams involve playing practice games between different groups of athletes participating in the team practice. In other cases, the researchers asked their participants, such as SCRABBLE players (Halpern and Wai, 2007), to give a single estimate for how much time they played and practiced per week. Some of the investigators collected estimates for how much time participants spent playing games as well as the amount time spent in team practice, which allowed them to assess the relative impact of participating in these different types of practice activities, but many did not.

### Quantification of the Amount and Quality of Practice Accumulated During the Entire Period of Development

**FIGURE 2:**
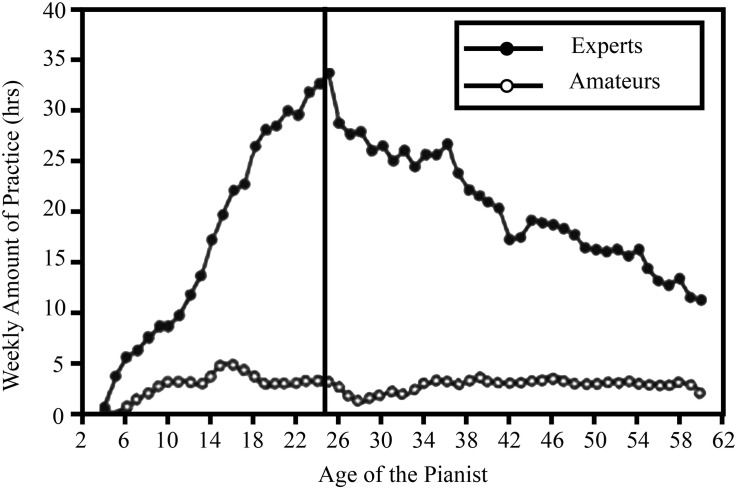
Pianists’ retrospective estimates of their weekly practice as a function of age. Data in the left panel are aggregated for young and older pianists. Data points above the minimum ages (20 for the young pianists and 52 for the older pianists) include at least 50% of the participants in each group (reproduced with permission of Figure 7 in Krampe and Ericsson, 1996).

It is challenging to recall and estimate the practice activities that expert performers have engaged in during their development of performance to an elite level. In most domains of expertise the development of elite performance may span a period of 5–30 years, and elite performers often attain the highest performance of their careers sometime between ages 15 and 40 (Ericsson and Lehmann, 1996; Haugen et al., 2018). Consequently, performers competing at the international level-have often accumulated between 3,000 and 40,000 h of engagement in domain-specific activities before they reach their highest level of performance (Baker and Young, 2014). The amount, quality, and specific type of practice activities will change dramatically from the time a child first engages in a domain until they reach the highest levels of performance. Ericsson et al. (1993) found that music practice is frequently organized on a weekly schedule, where the trainee meets with the teacher once a week and then practices at a regular time each weekday. With increased skill the trainee gradually increases the duration of their daily practice time from about 15–20 min a day to 4–5 h at the music academy. The high degree of organization of practice makes it possible for musicians to give reasonable estimates of their weekly amount of practice for each year of their development. Ericsson et al. (1993) explicitly focused on music students who were working full-time on improving their performance as their primary goal, and in a subsequent study Krampe and Ericsson (1996) extended their work to study amateur and professional pianists of ages between 52 and 68 years old. They found that time taken for solitary practice decreased after graduation from the music academy due to professional obligations involving public music performances and giving music lessons to music students, as is illustrated in Figure 2.

Krampe and Ericsson (1996) found that the older expert pianists, when they kept practicing around 10–15 h per week, were able to match the performance of the young expert pianists on tasks that were representative of music performance. The average number of hours of accumulated practice alone for the older expert pianists was 57,739 (*SD* = 20,159) compared to only 17,927 h (*SD* = 6,615) for the young experts (Krampe, 1994). In spite of having accumulated around 6 standard deviations more hours of practice than the young experts, the older experts’ performance was not superior. It was clear that the practice engaged in after graduation from the academy and the start of their professional career did not allow them to keep trying to improve their performance beyond its current level under the supervision of a teacher. Much of that practice focused on merely maintaining their already acquired performance, and Krampe and Ericsson (1996) referred to this practice as “maintenance practice.” They found that rather than using the accumulated practice over their entire career, a measure of their more recent practice activity was a better predictor of their current level of performance. If a musician stops playing music for several years it is well-known that he or she cannot return to playing their instrument at a high level of performance without first engaging in a lengthy period of practice. Krampe and Ericsson (1996) found that the accumulated amount of solitary practice during the last 10 years was the measure that best predicted individual differences in the participants’ performance on a range of experimental tasks, including when the analyses were restricted to the older and younger expert pianists. Similarly, Charness et al. (1996) noted that the current chess rating of older chess players was particularly influenced by their recent level of practice and was less well predicted by accumulated practice across their career when the predictability was compared to young chess players. We believe that the amount of accumulated practice necessary to reach the level of chess master estimated by Gobet and Campitelli (2007) is influenced by these issues. Gobet and Campitelli reported that the number of accumulated hours of solitary practice ranged from 1,612 to 14,196 to reach the level of chess master – a level corresponding to about the top 1% of the tournament players in a given country. We think that part of the variability in those numbers is likely influenced by whether the players remained fully committed to improving their chess skill throughout their career or whether they experienced periods of less intensive chess study where they play socially and only occasionally engage in solitary study. Similarly, Platz et al. (2014) noticed that Kopiez and Lee (2008) reported estimates for the accumulated number of hours of sight-reading music for both the period up to age 18 and also for the participants’ entire life. Platz et al. (2014) recommended using the first estimate, as this estimate is not confounded by increases due to age without documented efforts to improve. Although the correlation with sight-reading performance was larger for this estimate, Macnamara et al. (2014) selected the life-long estimates with a lower correlation with performance. It is essential that future studies differentiate the periods when individuals are fully committed to reaching their highest levels from those periods when they are primarily engaging in practice to maintain their performance or periods when they stop engaging in the domain.

The problem of returning to one’s earlier level of expert performance after a period of inactivity has been examined in the domain of crossword solving (Moxley et al., 2015) and in the domain of exceptional memory (Yoon et al., 2018). It should be possible in future research to elicit the goals for the solitary practice activities during different periods of an individual’s life in order to allow the identification and analysis of the periods of purposeful practice as distinct from maintenance practice. The quality of the practice is more difficult to assess than the quantity. There is now research showing that the act of practice by oneself is not necessarily effective learning and thus would not lead to improvements in attained music performance. The solitary practice of many beginning music students does not involve goal-directed efforts to change (improve) their performance. Video recording of solitary practice sessions of children between 7 and 9 years of age showed that these children were not able to recognize mistakes and they simply played through the assigned music piece a few times, essentially without any improvements (McPherson and Renwick, 2001). The individuals’ motivation to improve makes a big difference, and Evans and McPherson (2014) found a significant correlation between the amount of practice and the attained performance only for children who reported wanting to master their instrument. In a large-scale survey of several thousand young (age 6–19) musicians Hallam et al. (2012) found that with an increased level of attained music performance (assessed by objective tests) musicians practiced for more time, used more effective practice strategies and relied on the use of a metronome and tape recordings of their music practice and public performance.

There is another issue related to measuring only the duration of training. For example, when athletes develop strength and power they engage in a small number of trials of near-maximal effort. This type of practice can only be executed for short duration until muscular fatigue sets in. As a consequence, researchers have found that in sports requiring sprinting and explosive power for short periods there is a more important effect of very high intensity compared to the duration of sessions with purposeful practice and attained performance (MacInnis and Gibala, 2017). More generally, there are many sporting events where it is more important to generate maximal intensity for short periods of time, which can be monitored with physiological measures, such as heart rate, rather than increasing the duration of practice with a lower intensity (Mujika, 2010). In those cases, individuals can benefit by engaging in these activities more frequently during the week rather than extend the duration of individual sessions.

Finally, when expert performers are interviewed many years or even decades later about their practice during their entire development, they likely have difficulties recalling individual sessions and are forced to estimate and infer the number of hours of engagement in domain-specific activities based on their daily schedules. In many domains, individuals engage in organized training activities in team sports and other sports directed by coaches and teachers. In some domains, individuals establish daily schedules when they have plans to engage in practice every weekday for a certain amount of time, but they would occasionally need to change their plans to accommodate the need to seek a doctor, a dentist, or other sorts of interruptions to training schedules. This pattern was observed for the musicians at the music academy by Ericsson et al. (1993). These musicians estimated their typical weekly practice alone by multiplying their daily predicted practice time. These estimates correlated [*r*(28) = 0.74] with the time for this type of practice derived from their daily diaries filled out each day for a subsequent week. Interestingly, the musicians also estimated their weekly time for leisure, but these estimates were not significantly correlated [*r*(28) = 0.082] with the time for leisure derived from diaries.

One of the most ambitious attempts to correlate durations based on weekly diaries and estimates of weekly engagement was conducted in chess by de Bruin et al. (2008). They instructed their chess players to collect weekly diaries for three different weeks across the year. The diary data was converted to weekly estimates of serious chess study and was found to correlate [*r*(34) = 0.60] with the weekly estimate given for the current year. These findings suggest that the musicians and chess players were able to estimate the number of weekly hours of practice for the current year with correlations between 0.60 and 0.75.

Consistent with a distinction between practice and play, Hopwood (2015) found a reasonable reliability for purposeful practice in her review of recall of practice activities in sports, but substantially lower reliability for estimates of informal sporting activities. Ward et al. (2007) asked a subset of their participants to estimate their practice per year on two occasions. They found high reliability for estimates only for the most recent 5 years, but estimates for practice at longer intervals were not significantly correlated. More valid measures of error in estimation has been found for comparing athletes’ estimated hours of practice with estimates given by their parents. Baker et al. (2003) found a correlation of *r* = 0.59 (*p* < 0.05) between the estimates of athletes and their parents. There have not been any studies that have collected concurrent diary data on weekly practice for aspiring experts throughout their entire development. The findings suggest reliabilities in the 0.6–0.8 range for the last year or two. It is plausible that the reliability and accuracy of estimates of weekly practice for as much as 15–20 years earlier will be considerably lower. A reasonable estimate of the reliability of these practice estimates would therefore be 0.6, which will later be used for correcting the correlations for attenuation. There are other methodological differences that will influence the reliability and validity of estimates of practice. In the original study Ericsson et al. (1993) asked participants to estimate how much practice alone that they had engaged in for each year since they started playing a music instrument. This study and others using a similar methodology show that the engagement in practice changes dramatically over an individual’s career in the domain.

There are a number of studies that have not collected detailed information about participant’s practice during their entire careers. For example, Howard (2012) asked his participants responding to an internet survey on chess to give only two estimates of their weekly estimate for studying chess. One estimate was the average weekly study in the past year and the second question was: “How many hours per week on average have you studied chess since taking up the game seriously?” (Howard, 2012, p. 362). It would have been very difficult to give an accurate answer to that question when the engagement has varied substantially over the preceding decades, as illustrated by the professional musicians whose weekly average ranged between 3 and 32 h per week during different stages of their careers as shown in Figure 2. The average age of Howard’s participants was a little younger than that of the musicians, but the mean age was still 35 years old, and Howard (2012) provided no evidence that his participants could accurately estimate their average weekly engagement by a single number. Consequently, we will not include Howard (2012) data on practice in our meta-analysis.

More generally, the detailed nature and structure of the engagement in practice activities will be very difficult to recall accurately in detail many years later. These issues should be less problematic for practice activities meeting the criteria for deliberate practice in domains with an established curriculum that prescribes a particular progression of mastery. In these domains a teacher will guide the student to engage in deliberate practice during the entire development. As long as the teacher is skilled in assessing improvable aspects of the performance of a trainee and is able to prescribe effective training, we can assume that the recommended practice activities will be effective if the trainee follows the teacher’s instruction and engages in the practice activities with full concentration.

### Assessment of the Correlation Between Indices of Estimated Amount of Accumulated Purposeful and Deliberate Practice and Attained Reproducibly Superior Performance

**FIGURE 3:**
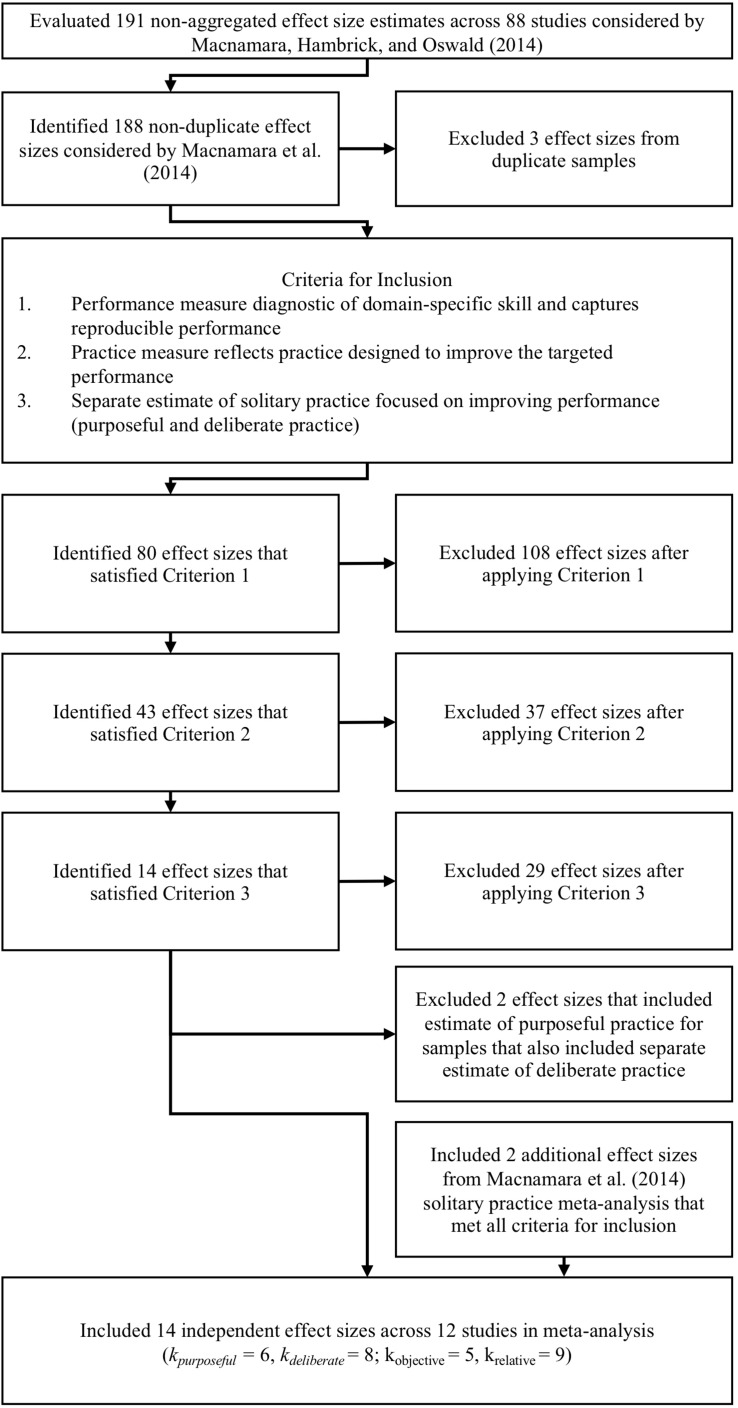
Flow diagram of applying revised inclusionary criteria to estimates of the effects of deliberate practice on performance considered by Macnamara et al. (2014).

In the previous sections we have discussed how studies collecting data on diverse types of performance measures and practice activities were included in Macnamara et al. (2014) meta-analysis. In this section we will attempt to specify explicit criteria for a subset of effect sizes included in their meta-analysis that can be included in a meta-analysis of the relation between accumulated purposeful and deliberate practice and the attainment of reproducibly superior performance in a domain of expertise. In our review we will be very conservative, and some of these effects could potentially have met our criteria if the investigators had included more information and reported information about different practice activities. First, the measures of performance used by studies included in the Macnamara et al. (2014) meta-analysis will be examined to find those that meet the first criterion that their dependent variable measured reproducibly superior performance in a recognized domain of expertise (see Figure 3).

#### Identification of Studies Measuring Reproducibly Superior Performance in Domain of Expertise

In an earlier section we discussed the problems with most of the studies on education included in Macnamara et al. (2014) meta-analysis. These studies measured performance by one or more tests in a course or by the students’ grade in the course. These measures are not acceptable as measures of reproducible performance in a recognized domain of expertise. When teachers assign grades in a course the grades are nearly always subjective judgments rather than an objective measurement of performance. Similarly, Macnamara et al. (2014) included effects from other studies where the performance variable consisted of ratings of athletes, musicians (Ruthsatz et al., 2008), and professionals (Sonnentag and Kleine, 2000). For example, Hendry (2012) and Memmert et al. (2010) relied on coach-generated ratings for assessing the performance of their players. There are clear problems with the validity of subjective ratings by a single person, especially if that person is responsible for deciding how much a given player will play during matches and other consequential decisions with regard to the players’ training. More generally, it is not clear how one can assess the reliability and, in particular, validity of those ratings of a given individual or even a group of individuals. It is possible and even likely that different coaches with similar, yet independent, knowledge of players would have given different ratings. In addition, it is essentially impossible to study athletes’ development of performance if we cannot directly compare ratings of different judges. The problem of comparisons is particularly salient if we want to compare performance across historical time, such as the present time versus 100 years ago, or across different countries, such as China and Sweden. Ratings are based on relative judgments of abilities and performance whereas other domains of expertise rely on measurements of absolute objective performance, such as time to run 100 m, number of strokes to complete a golf course, and the results of tournaments (Ericsson, 1996). In domains with absolute measurements it is possible to describe individuals’ performance by their level of competition, which would be primarily at the local, regional, state, national, and international levels. In that case there is a very close relation between the level of competition (a relative measure of performance) and the average performance of participants at the same level. In team sports, athletes’ performance is often inferred from the level of competition of their respective team. In those cases, it is less clear how differences between individual athletes in teams competing at different levels correspond to differences in absolute performance, which may depend on individual differences among players on the same team, such as the playing position within a team. In our meta-analysis we will examine the potential effects of the distinction between relative and absolute performance by including it as a moderator.

Some of the studies included in in Macnamara et al. (2014) meta-analysis failed to provide evidence for a reproducible superiority, such as Law et al. (2007), where only the performance at an Olympic competition was cited as evidence for the superiority of the Greek team of rhythmic gymnasts over the Canadian team. This is a case where the authors of that study could have been able to report evidence on reproducibility of the superior performance of the Greek team across many competitions in a season, but they did not. Our review assessed whether all studies and their associated effect sizes in Macnamara et al. (2014) meta-analysis met Criterion 1, which required that the dependent variable had to measure reproducibly superior performance that qualified as a measure of expertise in the associated domain.

#### Identification of Studies Where Practice Is Designed to Improve the Targeted Performance

According to the deliberate practice framework, goals for a desired level of performance should drive the design of training and practice to help trainees to reach that performance. Studies of practice within the expert-performance approach would therefore meet Criterion 2 and measure duration of practice activities that are motivated by and designed to attain a higher level of the targeted performance (see Criterion 1). This requirement would seem obvious based on the large body of evidence on the specificity of training effects (Reilly et al., 2009).

In some domains, such as music, it has been challenging to find measures of musicians’ ability to perform memorized music that can be administered easily and repeatedly during the year, in contrast to the use of juries at music competitions. Consequently, researchers have collected data on music-related tests involving sight reading, where a musician is asked to play an unfamiliar piece of music without opportunity to practice it. Sight reading is a very important activity for professional accompanists, but most music training focuses on helping musicians study a piece of music and then often memorize it. When ready, the musician would perform the piece of music with an orchestra for a large public audience. Macnamara et al. (2014) includes datasets that correlate the amount of deliberate practice toward becoming a soloist with the performance on tests of sight reading. Lehmann and Ericsson (1996) found that accumulated hours of deliberate practice was not significantly correlated with sight reading performance [*r*(14) = 0.32, *p* > 0.05], whereas the hours of accompanying performance was significantly correlated with this type of performance [*r*(14) = 0.630, *p* < 0.01]. In fact, when sight reading repertoire was included in the regression equation around 56% of the variance in sight reading performance was explained. Consequently, we will exclude effect sizes from studies relating amount of deliberate practice to performance on laboratory tasks, like sight reading tests, that do not explicitly capture the skilled performance that the individuals are training to attain. Platz et al. (2014) conducted a meta-analysis of a wide range of estimates of accumulated experience as well as estimates of accumulated deliberate practice on different measures of performance on sight-reading tests, and performance on laboratory tasks. Although the majority of the included studies measured accumulated experience, such as number of sight-reading performances, the aggregate relation between accumulated experience was impressive with a corrected correlation of *r* = 0.61 accounting for 36% of the variance in performance.

There are several other studies included in the meta-analysis where the accumulated practice estimates have been related to available performance variables without first demonstrating that the practice was directed toward improving each of those particular performance variables. For example, the accumulated practice estimates for the soccer referees in a study by Catteeuw et al. (2009) included many types of activities in their practice estimates. These researchers explicitly remarked that the hours of practice mostly were not relevant to improve skills related to accurate calls during games and tested scenarios. They recommended a search for practice activities that could include “additional decision-making experience outside match-play” (p. 1134).

In our review we examined all effects included in Macnamara et al. (2014) meta-analysis that had met Criterion 1, and assessed if the practice measure reflected practice directed toward improving target performance and that the estimate of accumulated practice accurately represented the sum of time spent engaging in practice activities that are directed toward improving the target performance (Criterion 2, see Figure 3).

#### Identification of Studies Where Estimates of Accumulated Practice Meets the Criteria for Measuring Solitary Practice Focused on Improving Performance (Purposeful and Deliberate Practice)

A common type of practice activity in many domains involves training in a group, often led by a teacher or coach. It is certainly possible that individuals are able to engage occasionally in training that would be most relevant to a given individual’s improvement during such training in groups. Based on the definition of deliberate practice we argue that the effectiveness of such group training would be inferior to a situation where the individual engages in solitary practice recommended by a coach or teacher (deliberate practice) or engages in solitary practice to attain a particular improvement determined by the individuals themselves (purposeful practice). In the solitary versions of the practice, the individual would be in full control of what to practice and for how long to engage in a particular practice activity.

All effect sizes included in Macnamara et al. (2014) meta-analysis that had met both Criterion 1 and 2 were examined to assess whether or not they provided an estimate of accumulated practice reflected time spent engaging in solitary practice or engaging in practice under individualized supervision of a coach or teacher (Criterion 3, see Figure 3). Nearly all effect sizes that were excluded relied on estimates of team practice or practice with groups of other individuals. For example, one of the included effect sizes referred to the study of Duffy et al. (2004) on dart players which included time spent practicing with a partner. Several other effect sizes were excluded because they included the time spent in team practice, such as a study of bowlers (Harris, 2008), of middle distance runners (Young et al., 2008), and of soccer players (Ward et al., 2007). The criterion was applied in a conservative manner so if the study did not request or report a separate estimate for solitary practice it was excluded. The general argument is that different practice activities might have differential effects, and in our review we are trying to assess the relation between the attained reproducibly superior performance and the accumulated duration of deliberate and solitary purposeful practice.

#### A Reanalysis of the Subset of Studies Included in Macnamara et al. (2014) Meta-Analysis That Meet the Three Criteria for Purposeful and Deliberate Practice

Our reanalysis of Macnamara et al. (2014) meta-analysis considered all of the effect sizes included in their analysis. First we eliminated effect sizes of two studies where the same study participants’ data were included twice as independent effect sizes extracted from two different articles as is shown in Figure 3. The first duplication involved Ureña (2004) dissertation data and the subsequent publication of the same data in an article under her married name, Hutchinson (Hutchinson et al., 2013). The second duplication concerned the data from Study 2 in Ericsson et al. (1993), which provided half of the data analyzed in Study 1 by Krampe and Ericsson (1996, p. 355). Macnamara et al. (2014, see Figure 2) found that the data from Ericsson et al. (1993) was reported as the 2nd highest effect size relating structured practice and performance, when reported for Ericsson et al. (1993). When essentially the same data was reported for Study 1 in Krampe and Ericsson (1996), it was reported as the 155th highest (3rd from bottom) for the experts and 130th highest (28th from bottom) for the novices. In Supplementary Text S1 (see Supplementary Data Sheet S1 in the Supplementary Material), we describe the reason for this remarkable reduction of the effect size, which is due to a separate analysis of experts’ and novices’ performance on a task designed for allowing performance by amateurs as opposed to analyzing all participants simultaneously. These three duplicate effect sizes were excluded from further analysis.

We then applied the first, second, and third criteria for inclusion sequentially and report the number of effect sizes that met that criterion in Figure 3. In Supplementary Data File S2 (see Supplementary  Data Sheet S2 in the Supplementary Material), we provide a listing of the inclusion or exclusion status for each of the effect sizes considered, and we describe the rationale that led to exclusion of each rejected effect size.

**FIGURE 4:**
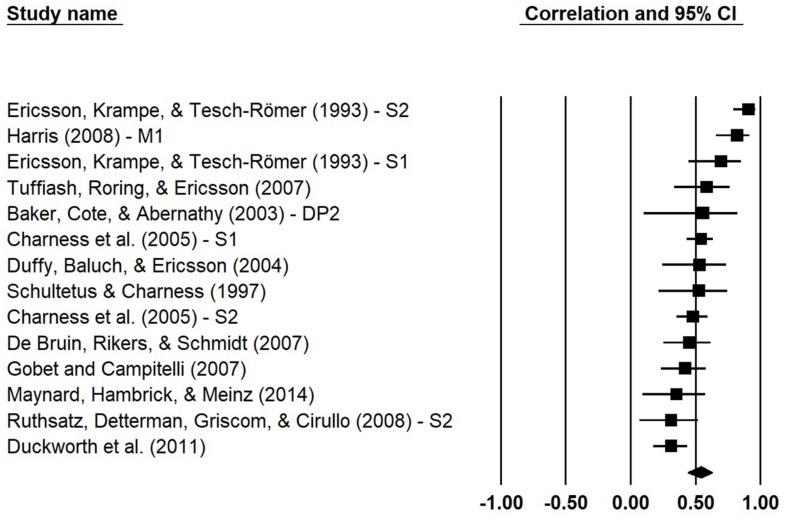
Correlations between purposeful or deliberate practice and performance. Squares represent correlation coefficients for the effects of practice on performance for each listed sample, and lines represent 95% confidence intervals. The marker at the bottom shows the weighted mean correlation. Study naming conventions were kept consistent with those used by Macnamara et al. (2014) for comparison purposes, indicating separate samples (e.g., S1) or measures of practice (e.g., DP2) or performance (e.g., M1).

Once the set of effect sizes had been identified as meeting all three criteria, we coded these effect sizes for two dichotomous moderator variables. The first represented objective versus relative measurement of performance, based upon whether performance estimates were derived from objective measurements or membership in groups of differing skill levels. The second moderator variable denoted whether the solitary practice estimates represented deliberate practice, where time was spent engaging in individualized practice activities according to the instruction of a coach or teacher or purposeful practice, and where the individuals were not guided by a coach. More detailed information regarding the procedure for study selection and moderator coding can be found in Supplementary Text S1, and a list of the selected studies and their effect sizes can be seen in Figure 4. It is worth noting that in their original analyses Macnamara et al. (2014) found significant moderator effects for the domain of performance, but due to the low representation of effects from some of the performance domains we could not replicate this categorical moderator analysis with our present selection of effects (see Fu et al., 2011). Sample-weighted means were calculated using the Comprehensive Meta Analysis software (Version 3.3, Biostat, Englewood, NJ, United States) for effect sizes from the domains of games (*r* = 0.50, *k* = 5), music (*r* = 0.71, *k* = 3), and sports (*r* = 0.58, *k* = 5). It is notable that no effect sizes from the domain of professions met the criteria (*k* = 0) and only the study of Spelling Bee performance (Duckworth et al., 2011) remained for the education category (*r* = 0.31, *k* = 1).

We used the Comprehensive Meta Analysis software to compute the random-effects weighted average of the selected effects. Results indicated a significant positive relationship between accumulated purposeful or deliberate practice and performance (*r* = 0.54, 95% CI = [0.44, 0.63], *p* < 0.001) accounting for approximately 29% of the variance, which is considerably higher than the 14% of explained variance reported in the analysis conducted by Macnamara et al. (2014). The first moderator analysis found that both objective and relative performance were significantly correlated with practice (*r*_*objective*_ = 0.49, *r*_*relative*_ = 0.65, *p*s < 0.001), with no significant difference between the two performance-type correlations [*Q*(1) = 1.45, *p* = 0.23]. This suggested that the positive relationship between practice and performance was not dependent upon whether performance was evaluated through group membership or objective measurement. The second moderator analysis indicated that practice was positively associated with performance whether it was conducted under the guidance of a coach or teacher (*r*_*deliberate*_ = 0.56, *p* < 0.001) or not (*r_*purposeful*_* = 0.51, *p* < 0.001). This difference was not statistically significant [*Q*(1) = 0.22, *p* = 0.64].

Finally, we also conducted separate meta-analyses comparing the effects of purposeful or deliberate practice on performance with the effects of structured practice for the subset of eight studies that reported separate estimates for both naïve practice and either purposeful or deliberate practice. Results indicated that purposeful or deliberate practice was more strongly correlated with performance (*r* = 0.51, *p* < 0.001) than was naïve practice (*r* = 0.39, *p* < 0.001), although the dependency of the samples and their practice measures precluded us from formally testing whether this difference could be generalized. Future research with independent training groups will be needed to precisely quantify the differences and test their significance statistically.

#### Correcting Our New Estimate of Accounted Variance for Attenuation

In this paper we have reviewed the evidence questioning the assumption that a single sum of the accumulated hours of practice is a theoretically valid predictor that would be able to account for the majority of individual differences in attained performance in a domain. Although we don’t accept the hypothesized theoretical relation between a single sum of hours of practice and attained performance, we would expect a correlation between more hours of purposeful and deliberate practice aimed at improving some specific aspect of performance and observed increases in the related performance. Both Hambrick et al. (2014) and Macnamara et al. (2014) clearly state that identified correlation between accumulated practice and performance must be corrected for lack of reliability in both the predictor and the performance measure. In an earlier section we reviewed evidence on reliability/validity of the estimates of practice and found that an estimate of 0.6 would be an appropriate measure for estimates of practice over prior years and decades. The reliability of the performance measure was discussed by Hambrick et al. (2014), who found a high level of reliability for chess ratings of international level players. In an interesting analysis, Glickman and Jones (1999) measured the reliability of ratings of chess players and found similar high estimates of Cronbach’s alpha equal to 0.95 for highly-rated chess masters but substantially lower Cronbach’s alphas equal 0.59 and 0.65 to for players in the average range between 1200 and 1800. Glickman and Jones (1999) hypothesized that the difference was due to the typical players being less involved in tournaments and chess activities. There are surprisingly few estimates of the reliability of performance measures for the samples used in studies of expert performance. Malcata and Hopkins (2014) reviewed research in sports and found intraclass correlation coefficients (ICCs) for athletes’ performance within a season and across a year ranging dramatically across domains. For example, cross-country skiing had an ICC for within-season practice of 0.93 and across a year of 0.9, whereas triathlon had ICCs of 0.36 and 0.15, respectively, and canoe slalom had ICCs of 0.31 and 0.27, respectively. Clark et al. (2008) calculated Cronbach’s alpha for amateur and professional golfers’ 18-hole rounds and found them to be 0.53 and 0.69 respectively. The reliability of performance seems to be higher for the most skilled performers in these diverse domains, but it is clear that it is well below one. Based on the available information we suggest that a reliability of 0.8 would a reasonable estimate. Following Hambrick et al. (2014) recommendation we can now correct the variance estimate of 29% by the lack of perfect reliability in the practice estimates (*r* = 0.6) and for measures of performance (*r* = 0.8). After correction for attenuation, our estimate for variance accounted for by the accumulated estimates of deliberate and purposeful practice is now 61%. These estimates are consistent with recent studies that show that a larger set of variables describing the practice history of individuals can account for over 50% of variance in chess (Burgoyne et al., 2019) and in SCRABBLE (Moxley et al., 2019).

All of the samples of individuals analyzed in the meta-analyses relating accumulated practice and attained performance only examine data from individuals who exhibit an acceptable level of skill. For example, even amateur players need to have played a lot of chess before they have engaged in a sufficient number of chess tournaments to be given a personalized chess rating. When samples are selected in a manner that is correlated with variables studied – namely, a minimal level of attained skill – then investigators correct for the restriction of range, which estimates substantially larger correlations for the entire population (Schmidt et al., 2008). Macnamara et al. (2016) emphasized (even including the finding in the abstract) that when they limited correlations only to elite performers, who had a substantially higher cut-off for their performance to be included, this estimated correlation, when compared to correlations for samples with a mixture of performers at different levels (amateurs, regional, and international), was much smaller and did not reach significance. Macnamara et al. (2016) do not even mention that this finding is completely consistent with the severe restriction of range. Unfortunately, none of the studies of only elite samples analyzed by Macnamara et al. (2016) passed our three criteria, so future research is necessary to assess if the relation between estimated duration of accumulated purposeful and deliberate practice is significant when only elite performers are included in the analyses.

### Beyond Correlations of Sums of Accumulated Duration of Practice and Attained Reproducible Performance

Our review has, so far, primarily attempted to show that imposing criteria for studies before including their correlations in a meta-analysis measuring the relation between accumulated amount of purposeful and/or deliberate practice and attained reproducible performance led to higher correlations than those found for *structured practice* by Macnamara et al. (2014, 2016). As discussed earlier in this paper, nobody has argued that any single hour of practice has an equivalent effect on improving performance. Consequently, we would not expect that completely error-free measures of accumulated practice and performance for the entire population of individuals would be perfectly correlated. Macnamara et al. (2014, p. 1608) reported that their estimates of the correlations between practice and performance were lower than expected, and that “deliberate practice is important, but not as important as has been argued.” In contrast, we argue that the current knowledge of the relation between quantity and quality of practice and resulting improvements in performance is steadily increasing as we distinguish the effects on performance from engaging in different types of practice activities, but it is still rather limited. In an earlier section we showed that the duration of effective training is not related to hours of engagement in practice activities for developing the strength and endurance of expert athletes, but the critical aspect of training is the intensity of the practice (Mujika, 2010). Similarly, we reported evidence that some students can engage in solitary practice without improvements in performance (McPherson and Renwick, 2001), and that strategies for improving performance increase in complexity as the attained level of performance is higher (Hallam et al., 2012). There is also an increasing body of research showing that increases in performance as a function of further practice are often not monotonic and exhibit plateaus in the individuals’ performance (Gray, 2017), which are not unmodifiable and can be overcome by changes and coach-led practice (deliberate practice). More generally, the development of an individual’s performance will be influenced by the quality of acquired basic skills and mental representations. The development of a particular individual’s performance requires intermittent assessment of skills, physiological adaptations, and mental representations, along with measurement of objective reproducible performance capturing expertise in the particular domain (Ericsson, 2018a,b,c). Only future research documenting the detailed history of practice and associated improvements of performance and mediating mechanisms will lead to significant advances of our understanding of the potential limiting factors of individual differences in innate ability that constrain the development of superior performance in a domain.

## Inferring Genetic Limits for the Effects of Practice on Attained Performance

An attempt to measure upper limits of improvability through practice will never be established by correlating a single measure of hours of accumulated practice with attained performance. It is therefore important to pursue an alternative approach which would involve identifying those anatomical and physiological characteristics that *cannot* be changed by practice, diet, or other environmentally controllable factors.

In the original paper, Ericsson et al. (1993) readily acknowledged that there are individual differences in characteristics that are correlated with attained performance yet cannot be modified with any known type of practice. For example, this paper mentioned that research on the development of height and body size (differences concerning the length of bones) indicate that they are determined by genetic factors. This paper even offered some evidence suggesting that there might be inherited factors that influence an individual’s ability and willingness to sustain the focus and concentration necessary for successfully engaging in deliberate practice. Even more importantly, this paper reviewed evidence proposing it is possible to dramatically change most anatomical and physiological attributes by engaging in particular types of practice, in contrast to the genetically determined height and body size.

Most of the scientific knowledge about the degree of influence of genetic factors has been based on studies of twins and the degree to which identical twins are more similar than fraternal twins in a wide range of attributes. The most cited measure of genetic influence is heritability, namely the percentage of variance in individual differences of some measured performance or characteristic that can be accounted for by genetic factors by comparing individuals with differences in the degree of genetic similarity, such as twins and family members. It is, however, important to recognize that “heritability describes ‘what is’ in a particular sample; it does not connote innateness or immutability,” in the words of some of the most influential behavior geneticists (Plomin et al., 2014, p. 47). This implies that we should not assume that heritability estimates for various measures of physical performance of individuals who lead mostly sedentary lives with engagement in mostly recreational physical activities are valid heritability estimates for expert performers, who have engaged in extensive training for years and even decades. In fact, Plomin et al. (2014) agrees and argues that we should consider expert performance as “what could be.” The extensive body of research (Ericsson, 2014) shows that expert performance is mediated by acquired cognitive skills and physiological adaptations which differ from those available to beginners. These considerations imply that we should not use heritability estimates derived from novices or amateurs in a domain to reflect the corresponding heritability estimates for individuals who have an extensive training history and perform at a very high level.

There is a large body of twin research that has assessed heritability of scores on tests measuring characteristics believed to be important for success in sports, such as physical fitness, fast-twitch and slow-twitch muscle characteristics, and degree of body fat. These heritability estimates suggest a substantial influence of genes, which has led some researchers (MacArthur and North, 2005) to propose that inherited genes will be the most important factor for predicting elite status of athletes. An early review (MacArthur and North, 2005) suggested that a single gene would explain some 20–40% of individual differences in each of these physical characteristics, such as strength, power and endurance. In the last decade new technological advances have made it possible to describe all the genes in individuals’ genomes and do so for many thousand athletes and non-athletes. Genome-Wide Association (GWA) studies have analyzed all this information to search for those particular genes that are associated with a particular superior performance. A recent general review concluded that the genes identified with GWA studies accounted only for a minor fraction of variance predicted by the twin studies (Eynon et al., 2017). So far there appears to be no single gene that accounts for even a few percent of the variance in any of the athletic characteristics (Moran and Pitsiladis, 2017). Even when GWA studies have searched for unique genes in very popular sporting events, such as endurance running, not even a single gene was found to consistently predict significant differences between world-class runners and sedentary adults (Rankinen et al., 2016).

There are many possible explanations for this discrepancy (Georgiades et al., 2017). Most twin studies have collected data on twins who led normal lives and thus had not engaged in intense training necessary to attain elite performance levels. This observation has raised issues about the generalizability of heritability estimates based on the original twin studies (Ericsson, 2014; Georgiades et al., 2017). Another issue is that the similarity of identical twins’ physical fitness reflects both their identical genes and the similarity of their engagement in physical activity and potential interactive effects. One interesting approach to distinguish these influences is to search for identical twins where one member of the pair has been engaged in physical activity and the other has been sedentary. Leskinen et al. (2010) identified ten twin pairs meeting those criteria and found reliable differences in the degree of expression of genes in cells of muscles and other tissues consistent with the differences in maximum oxygen consumption and amount of fat. In a recent case study, Bathgate et al. (2018) compared the physical characteristics of a track coach, who participated in many marathons, to his identical twin, who was a truck driver with a sedentary life style. The active twin had dramatically different physiology with a greater maximum oxygen uptake (over 20% higher) and much slower twitch fibers (55% more). The two twins had comparable life styles until age 20, but their lives diverged for the subsequent 30 years. If the track-coach twin had engaged in training typical of elite athletes during childhood and adolescence it is likely that the differences between the two twins would have been even larger. In a large sample of twins, Eriksson et al. (2017) interviewed ten twin pairs reared together, where only one of the identical twins was currently an amateur playing a keyboard instrument and had practiced more than 1000 h more than the other twin, who was not playing an instrument. Eriksson et al. (2017) was unable to identify any systematic environmental factors that could explain the discrepancy. When these twin pairs, except one, had their brains scanned, De Manzano and Ullén (2018, p. 392) found “that even when controlling for genes and early shared environment, there can be observable neuroanatomical differences in both gray matter and white matter microstructure between individuals that differ vastly in musical training.” The authors furthermore speculated that the differences between the two identical twins would have been even larger if the music playing twin had been a professional musician rather than an amateur.

Twin research on cognitive ability has also estimated that a substantial portion of the individual differences in performance of tests measuring cognitive ability is heritable (around 50%; Plomin and Spinath, 2002). It has been assumed that superior cognitive ability would be associated with superior performance in domains of expertise across the entire period of development of expert performance. In a review, one of us (Ericsson, 2014) showed, however, that the performance of beginners in a domain of expertise correlates with scores on tests of general cognitive ability, whereas the performance of skilled individuals in the same domain correlates with such test scores at a dramatically reduced level and often cannot be distinguished from chance. In a subsequent review, Ullén et al. (2015) mentioned two studies that would still show significant correlations between performance on tests of general cognitive ability and performance. They cited a significant correlation between amount of deliberate practice for traditional music performance and performance on a test of working memory and sight-reading performance (Meinz and Hambrick, 2010). Consistent with our earlier described criteria for examining only performance that captures the goal of the music training, we will not discuss this finding further. More importantly, they also cited a significant correlation between intelligence and chess ratings (Grabner et al., 2007). However, in a more recent meta-analysis of the correlation between cognitive-ability tests and chess performance, Burgoyne et al. (2016) found a substantial correlation between test scores of cognitive ability and chess performance for beginners and less-skilled players, but the relations were no longer significant for highly-skilled players. There is an accumulating body of evidence for a gradual disappearance of correlations between performance on cognitive ability tests and domain-specific performance as domain-specific mechanisms are acquired and then mediate the superior expert performance.

Some recent studies have analyzed large samples of identical and fraternal twins to assess the heritability of attained performance in domains of music. Hambrick and Tucker-Drob (2015) examined data on twins among high-school students and found that having engaged in some type of public music event, such as at a minimum receiving a good evaluation at a music competition at the school level, was significantly heritable. When we reanalyzed this data set while defining the music achievement matching the students at the music academy in West Berlin (Ericsson et al., 1993), the estimate of the additive genetic effect was no longer significant (see our Supplementary Text S1 for details). In another very large sample of over 10,000 twins, Mosing et al. (2014) tested twins on a test of music ability and estimated substantial heritability (40–70%). Mosing et al. (2014) proposed that “results may have differed if a different measure of music ability had been used (e.g., success in the musical world)” (p. 1802). Consistent with the possibility that heritability estimates would not be significantly different from zero when success in the music world was defined as becoming a successful professional musician, the number of musicians that had reached a professional level was reported to be very small (Ericsson, 2016). In response to a request to Mosing and Ullén for an ACE analysis of the effects of genetics on reaching expert-level musical performance (professional status, in this sample) sent shortly after the study was originally published, the authors responded that they were going to publish these results very soon. Now 5 years later, and after many repeated requests for such an analysis there has been no such reporting on the professional musicians in their sample. This group of researchers has published several papers on twins where only one identical twin in a pair is playing music but they limited these analyses to the amateur musicians in their sample (Eriksson et al., 2017; De Manzano and Ullén, 2018).

More generally, Ericsson (2014) reviewed the information of the elite achievement of twin pairs or individual twins of either identical or fraternal type. The review uncovered very few cases, in fact a much smaller number than would be expected by chance based on the proportion of twins in the general population. It is therefore unlikely that studies of identical and fraternal twins will ever provide us with information relevant to estimating the heritability of attaining expert performance.

## Toward Future Integrated Accounts of Individual Differences in Attained Expert Performance

The expert-performance framework and the proposals by Hambrick et al. (2014), Macnamara et al. (2014) and Ullén et al. (2015) have many agreements. All of them agree that extended practice is necessary to attain expert performance and that genes in the DNA are expressed in response to practice activities, and these genes play a central role mediating the biological changes of body and nervous system. All frameworks also agree that unique genes generate individual differences that are important predictors of successful performance in some domains, such as height in many sports, and that future research in genetics might identify unique genes related to success in various domains of expertise. Our disagreement with Macnamara et al. (2014) and Ullén et al. (2015) concerns their claims of having uncovered limits for how much performance can be improved by practice, in particular that Macnamara et al. (2014) reported limits generalize to purposeful and deliberate practice. Only future empirical research will allow us to describe and measure these limits and then assess whether these potential limits will practically constrain some individuals from attaining expert performance in particular domains.

There are suggestions that future research will be better integrated and combine the two types of traditionally unrelated studies. The first type of traditional research consists of studies analyzing only the GWA of genes to superior performance. The second type focuses on analyzing cognitive mechanisms and detailed analysis of engagement in practice activities and the changes in performance resulting from engagement in these practice activities. Over the last few years geneticists (Georgiades et al., 2017) have expressed the goal of explicating epi-genetic effects on performance, and they propose collecting information about the detailed practice history along with genome-wide mapping of genes so that practice activities involving parts of the body that trigger the expression of genes in corresponding tissues can be identified.

In the future it should be possible to analyze the individual differences in attained absolute performance in a particular domain with regression analysis, where variables include the presence of unique genes, the engagement in particular practice activities, as well as the possible interaction between genetic and practice variables. It is less clear that proposals for including predictors such as measures of personality and general cognitive ability (Ullén et al., 2015), will be particularly helpful in assessing the relative role of the genes, practice and their interactions, as it is currently clear that it is impossible to infer whether these individual differences in general cognitive abilities (Habibi et al., 2018) or personality (Tedesqui and Young, 2017) are the cause or consequence of the extended engagement in practice and instruction. A major challenge to a regression approach to identifying the predictors of very high levels of performance is that the number of individuals meeting the standards of absolute performance at the highest level is small in each particular domain of expertise. It is therefore unlikely that we can successfully induce knowledge by collecting data from thousands or millions of participants and then use statistical techniques to infer which of the many study variables can predict performance outcomes for new samples using cross-validation methods. This is a well-documented problem for GWA studies that evaluate the potential effects of a very large number of genes (Rankinen et al., 2016) where the probability of spurious associations between genes and performance are high. Earlier in this paper we discussed problems of reducing the variables measuring practice to a single sum of the accumulated hours of engagement in many types of practice activities. In fact, when a larger number of variables measuring different aspects of the practice history is entered into the analysis the amount of explained variance will increase. For example, a recent reanalysis of practice-related variables (Burgoyne et al., 2019) showed that they accounted for over 50% of the variance in chess ratings, even without any corrections for attenuation. In our opinion, we are unlikely to be able to account for all individual differences in attained performance that would be attributable to practice and training. By incrementally including variables measuring at what age certain types of practice activities were initiated and how many hours an individual engaged in certain type of activity, as well as the observable result of that practice, the amount of variance accounted for will slowly increase. This approach might be pursued by some researchers, but it will not address the questions originally motivating our research on expert performance (Ericsson et al., 1993).

The original goal of the *Psychological Review* paper (Ericsson et al., 1993) was to search for and then describe optimal training conditions for improving the reproducible objective performance in domains of expertise. The first two studies examined the daily lives of full-time students in the domain of music, which has had a very long history of developing one-on-one training and thus developed effective practice methods and a common curriculum for students. Within the music academies, students receive training consistent with the definition of deliberate practice, where a teacher assesses the individual trainees, provides guidance for their work during their solitary practice and evaluates their improvement related to the assigned practice goals at weekly lessons. Many domains of expertise can learn something from the training developed in music academies. To make further progress researchers need to go deeper and describe the quality of deliberate practice by examining the cognitive processes mediating effective learning during solitary practice (Coughlan et al., 2014) and/or analyzing detailed behavior during practice from video recordings and performance tests (Miksza, 2015). It is important that researchers objectively describe the structure of the acquired performance by each trainee as well as the processes of their skilled teacher, who assesses that performance and uses this knowledge to guide the trainee’s future practice goals. More generally, we would recommend that researchers invite trainees and their teachers or coaches to study the long-term development of absolute performance in the associated domain. In the last few years there are several reports describing the training and performance of World-class athletes in cross-country skiing (Solli et al., 2017), in Nordic combined skiing (Rasdal et al., 2018), and cycling (Pinot and Grappe, 2015). These studies have collected detailed information about each training session, often by downloading data collected during the practice sessions by a device (Pinot and Grappe, 2015) or data entry after each training session (Solli et al., 2017; Rasdal et al., 2018). In all these three cases, dramatic improvements of absolute performance were recorded during the examined time period of 5–10 years, and these changes were closely linked to changes in the duration and/or intensity of particular types of training.

In sum, we believe that a partnership between researchers and individual elite athletes and their coaches would allow relatively unobtrusive documentation of the detailed practice conditions along with the associated changes in performance on the practice task and associated verbal reports of thought processes during learning. This arrangement is very similar to the early research on memory experts, who were brought into the laboratory for extensive testing followed by experiments designed to evaluate hypotheses about the mechanisms mediating the experts’ superior performance (Ericsson, 2018a). The primary difference would be that these new studies would focus on expert individuals who are focusing on improving their performance for longer periods. The proposed collaboration should only minimally interfere with the trainees’ regular schedule because the researchers would record the data very unobtrusively during practice sessions and then analyze that data as well as invite the athletes to participate in occasional tests of performance in the researchers’ laboratories. Based on their analyses, the researchers will propose hypotheses about the cognitive and physiological mechanisms mediating observed improvements of absolute performance, which could subsequently be evaluated by designed experimental sessions with the trainees. The findings from the analyses and experiments will not merely improve our understanding of the conditions of optimal practice. For individual expert performers this arrangement should be beneficial because it would likely provide the financial resources from foundations, granting agencies, and sponsors to the researchers to conduct regular assessments and analyses of changes in performance as well as associated changes in mental representations, physiological adaptations, and perhaps even the associated expression of particular genes in relevant tissues. The accumulation of this type of knowledge will not only benefit teachers and coaches and their trainees in the same domain, but it will also allow scientists to induce general principles for how traits and mechanisms mediating competitive performance can be effectively modified to improve the performance of children, adolescents and adults in a wide range of domains of activities during work and leisure.

## Author Contributions

KE contributed conception of the review and wrote the draft of the manuscript. KH prepared the data, performed the statistical analysis, contributed to sections of the manuscript relevant to statistical analysis, and prepared Supplementary Materials. All authors contributed to manuscript revision, read and approved the submitted version.

## Conflict of Interest

The authors declare that the research was conducted in the absence of any commercial or financial relationships that could be construed as a potential conflict of interest.

## Supplementary Material

DATA SHEET S1 Supplementary Text S1, which provides more details regarding our meta-analytic procedure and reanalysis of the dataset analyzed by Hambrick and Tucker-Drob (2015).

DATA SHEET S2 Coding for inclusion and exclusion of effect sizes reported by Macnamara et al. (2014).
