# Supplementary material for: Deliberate Practice and Proposed Limits on the Effects of Practice on the Acquisition of Expert Performance: Why the Original Definition Matters and Recommendations for Future Research
Source: Front Psychol. 2019 Oct 25;10:2396. doi: 10.3389/fpsyg.2019.02396 (PMC6824411; doi:10.3389/fpsyg.2019.02396)
Supplement: DATA SHEET S1 — Supplementary Text S1, which provides more details regarding our meta-analytic procedure and reanalysis of the dataset analyzed by Hambrick and Tucker-Drob (2015). [file Data_Sheet_1.docx]

Supplementary Material

# Procedure for Meta-Analysis of the Effects of Deliberate or Purposeful Practice on Performance

The objective of this meta-analysis was to determine the relationship between accumulated amounts of deliberate or purposeful solitary practice and level of attained reproducible performance. The previously reported meta-analysis conducted by Macnamara, Hambrick, and Oswald (2014) included a number of effects that were calculated using practice and performance estimates that are inconsistent with the original definition of deliberate practice. In this study we sought to reassess the relation between performance measures that adequately captured differences in skilled performance, on the one hand, and practice measures that were sufficiently representative of deliberate or purposeful practice, on the other hand. We applied three necessary criteria when reevaluating all effects included in Macnamara et al,’s (2014) meta-analysis for inclusion in our analyses, and we kept each effect size that met the criteria for deliberate and purposeful solitary practice according to the expert performance framework (Ericsson, 2018; Ericsson, Krampe, & Tesch-Römer, 1993). First, the performance measure corresponding to each effect size must have been sufficiently diagnostic of differences in domain-specific skill and representative of reproducible performance. Second, the practice estimates must have reflected accumulated training directed toward improving the measured performance. Third, the practice estimates must have consisted of separate estimates of time spent engaging in solitary practice activities. Each of these criteria will be briefly discussed in more detail in the following sections.

The reanalysis of the meta-analyses reported by Macnamara et al. (2014) relied on a file with all the included data provided by these authors, downloaded from https://osf.io/rhfsk/. The data reported 203 effect sizes across 88 studies considered by the authors for inclusion, with a number of studies containing multiple effects that were aggregated into summary effects to bring the total number of included effects to 157. We evaluated all individual effects considered for the general analysis and did not include the aggregated effects or effect sizes that estimated effects for duplicate samples. The second author coded each effect size according to the criteria for inclusion sequentially, and the first author reviewed the coding and discussed all cases until consensus was reached. The second author then coded each of the included effect sizes for the moderator variables, after which the first author reviewed and confirmed the coding decisions. Finally, the second author reviewed the studies from which the included effect sizes were retrieved to identify separate estimates of accumulated engagement in naïve practice. Each case was discussed and confirmed with the first author.

## Preliminary Exclusion of Effect Sizes

Prior to applying our criteria for inclusion to the effect sizes considered by Macnamara et al. (2014), we excluded a number of effect sizes for technical reasons. First, we did not consider the 12 aggregated effect sizes calculated by Macanamara et al. (2014). These aggregated effect sizes represented combinations of multiple dependent measures and invariably combined effects across categorically different types of practice. Rather than considering these aggregated effect sizes, we evaluated the effect sizes that Macnamara et al. (2014) combined to calculate them individually and applied our criteria for inclusion to them independently. As such, there were situations where some effect sizes reported by a study met our criteria for inclusion while others were excluded due to violating one of the criteria. Additionally, we elected to exclude 3 effect sizes included in Macnamara et al.’s (2014) meta-analysis on the grounds that those estimates were calculated from duplicated samples, being already represented by effect sizes drawn from other studies. We excluded 2 effect sizes from Krampe and Ericsson (1996) which estimated the effects of solitary music practice on performance of a musical interpretation task for a sample of expert pianists and a sample of amateur pianists. This same sample of musicians was also represented by an effect size from Ericsson, Krampe, & Tesch-Römer (1993), Study 2. For that study, Macnamara et al. (2014) used group comparison of the amateur and expert musicians as the relative measure of performance, with the same estimate of solitary music practice as the measure of accumulated practice. We chose to retain the effect size based on comparisons of the two skill-groups from Ericsson et al. (1993) and excluded the 2 effect sizes from Krampe and Ericsson (1996) that investigated performance of a single task by the two skill-groups independently, as the former presents a better opportunity for capturing differences in the relevant domain-specific performance. The attenuated correlation between practice and attained performance was r= 0.906, whereas when Macnamara et al. (2014) established effect sizes for the relation between accumulated practice and performance on a music test for Study 1 by Krampe and Ericsson (1996) they estimated the relation separately for experts (r=-0.224) and amateurs (r=-0.004—but in their supplementary materials Macnamara et al. (2014) reported the effect size as 0.104), where neither correlation reached significance. If on the other hand, Macnamara et al. (2014) had measured the relation between the recommended measures for accumulated practice and that same test performance for the combined sample of experts and novices, then the raw correlation would have been r= 0.595, which would have been highly significant.

We also excluded the effect size retrieved from Hutchinson, Sachs-Ericsson, and Ericsson (2013), as the sample of dancers described in that study represented a subset of participants included in a study reported by Ureña (2004). Macnamara et al. (2014) included effect sizes for both studies. Given that the analyses from Hutchinson et al. (2013) describe the same participants from Ureña (2004), we considered the effect size retrieved from the later study to be a duplicate and did not consider it for the present meta-analysis.

## Criterion 1: Performance Measure Diagnostic of Domain-Specific Skill and Captures Reproducible Performance

The first inclusionary criterion was that the performance measure used to calculate the practice effect was representative of an accepted indicator of domain-specific skill and participants’ level of reproducible performance. Deliberate practice was originally proposed to explain one of the processes by which experts can efficiently acquire the knowledge and skills necessary to develop reproducible superior performance (Ericsson, 2018; Ericsson et al., 1993). Because deliberate practice is directed toward improving performance in a specific domain, it is important that comparisons of accumulated practice and attained performance use performance measures that are sufficiently representative of the specific performance domains individuals are working to improve. One way to do this is to rely on accepted indicators of domain-specific skill, or by classifying performers into groups of differing skill levels based upon these indicators. In ideal circumstances, the performance measures will involve multiple instances of completing standardized tasks so that performance can be objectively compared across different samples of performers. This objective comparison of skilled performance of representative tasks is critical when attempting to quantify the effects of deliberate or purposeful practice on performance. Importantly, it should be demonstrated that observed differences in performance are reproducible and stable, making it more likely they are the result of true differences in the relevant domain-specific skills and not a product of the sampling. A total of 80 effects met this criterion (2 from the domain of education, 8 from the domain of games, 13 from the domain of music, 1 from the domain of professions, and 56 from the domain of sports), and we will discuss a few examples of how we applied the criterion.

By far the largest departure from the analysis reported by Macnamara et al. (2014) was that we did not include the effect sizes that related accumulated hours of studying to a measure of academic achievement. This decision was motivated by previous research indicating that performance on tests of academic achievement are not accepted indicators of domain-specific skills. Additionally, the performance measures used by these studies typically represented a single instance of performance (i.e., course grade or performance on a single test), which does not meet the criteria for reproducible superior performance (Ericsson, 2018). Additionally, these studies of academic achievement are also problematic because they typically only gather information about the amount of studying participants engage in during the period of participation, and many participants will enter a given semester with some previously-obtained knowledge that is relevant to their current coursework. It is therefore unclear to what extent these performance measurements taken at a single timepoint are dependent upon previous studying behaviors or experiences not captured by the practice measures included in the effect sizes included in Macnamara et al.’s (2014) meta-analysis. Finally, as was discussed in the text, aggregated estimates of the highly irregular studying behavior that many students engage in tend to be poor predictors of academic achievement and do not meet the criteria for deliberate or purposeful practice (Plant, Ericsson, Hill, Asberg, 2005). For these reasons, we decided to exclude 65 effect sizes based on comparisons of estimates of general academic studying behavior to academic achievement performance measures.

We also excluded 25 effect sizes that were calculated using performance on laboratory tasks or other novel tasks that were used to approximate levels of domain-specific skills in the absence of accepted objective measurements of reproducible performance. These measures were designed to probe the specific research questions addressed by those studies by having participants complete tasks representing specific circumstances rather than from the broader domain of performance. The relationship between these measures and performance based on widely-used indicators of skill in those domains has not been established, making comparisons of domain-general skill across samples of participants difficult to interpret. In the case of Harris (2008), however, the study also reported a standardized measure of performance (bowling average), so we retained the effects relating practice to that measure of performance. We excluded 17 effect sizes from Berry, Abernethy, and Côté (2008), Hendry (2012), Memmert, Baker, and Bertsch (2010), Ruthsatz, Detterman, Griscom, and Cirullo (2008), and Sonnentag and Kleine (2000) associated with performance measures derived from subjective ratings of performance by a single rater. In all cases, the single rater was an authority figure (i.e., coach, band director, course instructor, or job supervisor) that was likely to have significant influence in making decisions regarding participants’ training experiences. It is also worth mentioning that the effect sizes from Berry et al. (2008), Hendry (2012), and Memmert et al. (2010) were calculated using coach-ratings of general abilities like creativity and decision-making applied to the sports context, which are not commonly-accepted indicators of sports performance. Finally, we excluded the effect size from Law, Côté, and Ericsson (2007) because the nature of the performance classification, at least as it was reported in the study, did not meet the criteria for reproducible superior performance. Participants were two teams of six rhythmic gymnasts that were classified as Olympic-level and international-level, based on their current world rankings at the time. The only information provided to differentiate the skill-levels of the two teams was that the Olympic-level team was “ranked second in the world in rhythmic gymnastics group competition at the time of the interview” and the international-level team was “ranked first in their country, but had never placed higher than 10th at the World level and were ranked 13th in the world standings at the time of study” (p.87). It is entirely possible that these world ratings were accurate representations of reproducible differences in performance; however, without more information regarding the competition history of the teams we were not able to make that determination.

## Criterion 2: Practice Measure Reflects Practice Directed Toward Improving Targeted Performance

Another important consideration when quantifying the effects of purposeful and deliberate practice on performance is that the estimate of accumulated practice must accurately represent the sum of time spent engaging in practice activities that are directed toward improving the performance. Out of all the time spent engaging in domain-relevant activities, only a portion of that time can be considered directly relevant for improving an individual’s skills. While many of the day-to-day activities people engage in could be indirectly associated with gains in performance, purposeful and deliberate practice activities are defined by having specific goals and are designed to improve a particular aspect of the targeted performance. It is also essential that the estimates of accumulated practice are elicited in such a way that they accurately reflect the practice history of the individual participants. A total of 43 of the remaining 80 effects met this criterion (education: 1, games: 5, music: 5, sports: 32).

We excluded 28 effect sizes from studies that included time spent engaging in naïve practice activities within their estimates of accumulated practice. For example, a few sports studies included structured practice activities like endurance training or playing sports other than the domain-relevant sport within their total accumulated practice estimates (e.g., Baker, Bagats, Büsch, Strauss, & Schorer, 2012; Helsen, Starkes, & Hodges, 1998; Hodges & Starkes, 1996). For Cathey (2010), which compared practice estimates for professional and amateur baseball pitchers, the practice estimate aggregated practice across pitching, fielding, and batting practice. Fielding and batting practice would have very little impact on improving pitching skill, so this effect was excluded. Similarly, we excluded one effect retrieved from Gobet and Campitelli (2007) because the practice estimate pertained to practice for chess but the performance measure pertained to speed chess; the rules differences between chess and speed chess necessitate different practice activities between the two games. As was discussed in the text, the effect from Howard (2012) was excluded because the author’s calculations of accumulated chess practice involved multiplying participants’ weekly estimates of chess practice by 52 and then again by the number of years elapsed since participants began competing in chess. This single, linear estimate assumes players’ chess study habits remained stable throughout their careers and is insensitive to the changes in practice frequency and duration associated with different stages of chess skill development. We also excluded 8 effect sizes relating accumulated solitary music practice to expert-rated music sight-reading performance (Kopiez and Lee, 2008; Lehmann and Ericsson, 1996; McPherson, 2005; Meinz, 2000; Meinz & Hambrick, 2010; and Tuffiash, 2002). Estimates of sight-reading performance do not necessarily reflect the acquired skill level of musicians preparing for performance of memorized pieces, and there was no reported separate measure of time spent engaging in solitary sight-reading music practice.

## Criterion 3: Separate Estimate of Solitary Practice Focused on Improving Performance (Purposeful and Deliberate Practice)

The majority of the effect sizes included in Macnamara and colleague’s primary meta-analysis were calculated using estimates of hours of practice that aggregated across a wide variety of practice activities. Many of these aggregated practice time estimates included structured practice activities where individuals completed them in a social context, either as part of a larger group of individuals also completing the same activity, or as competing with another player in a competitive sport or game. The definitions of deliberate and purposeful practice clearly indicate that practice should be individualized to allow the individual to focus on activities appropriate for developing his or her current skillset. More importantly, engaging in solitary practice maximizes the opportunity for the individual to receive immediate feedback about quality of his or her performance during the training task. Engaging in structured social practice activities may indeed be valuable for developing skill, but they do not conform to the definitions of deliberate or purposeful practice. Thus, we required practice measures consist of separate hourly estimates of practice that specified the practice was solitary (purposeful practice) or conducted under the individualized instruction of a coach or teacher (deliberate practice). In the few studies that provided estimates for both deliberate practice and purposeful practice, we retained the effect calculated using the accumulated amount of deliberate practice.

Macnamara et al. (2014) acknowledged the issue of the solitary nature of deliberate practice and conducted a supplementary meta-analysis where they examined the subset of effects where the authors felt the practice estimates were representative of solitary deliberate practice: “We tested this model to address the question of whether deliberate practice must be performed in isolation to be maximally effective” (p. 1615). They performed a separate meta-analysis including only the effect sizes they identified as corresponding to relationships between solitary practice and performance. We reviewed these effect sizes and identified studies where the solitary practice estimates differed from the practice estimates used to calculate the effect size included in the general meta-analysis. We coded those effects according to our criteria as well and included any effects that met all of our inclusionary criteria.

Of the 43 remaining effect sizes, 14 met this criterion. We excluded 29 effect sizes that were calculated using estimates of accumulated practice that included time spent engaging in practice activities with team or partners, the majority of which were from sports studies where accumulated structured team practice was considered by Macnamara et al. (2014) to be the deliberate practice measure. Two effects were included from the selection of studies included in the solitary practice meta-analysis that were not included in the general meta-analysis: one from Gobet and Campitelli (2007) and one from Duffy, Baluch, and Ericsson (2004). No information was available regarding Maynard, Hambrick, and Meinz (2014), but given the study was described as relating accumulated hours of solitary bowling practice to participants’ current bowling ratings, it seemed highly likely to meet our criteria and we decided to include this effect. Finally, two studies generated separate effects of both deliberate and purposeful practice on performance (Baker, Côté, & Abernathy, 2003; Schultetus & Charness, 1997), so we included only the effect sizes corresponding to the effects of deliberate practice. This resulted in a final total of 14 effect sizes to be included in the meta-analysis (education: 1, games: 5, music: 3, sports: 5). One effect from Baker et al. (2003) compared the amount of accumulated practice with individualized instruction of a coach until age 20 to group membership between national-level and less-skilled athletes. Two effects from two samples reported in Charness, Tuffiash, Krampe, Reingold, and Vasyukova (2005), and one effect each from de Bruin, Rikers, and Schmidt (2007) and Gobet and Campitelli (2007) related estimates of solitary chess study to participants’ current chess ratings. One effect from Duckworth, Kirby, Tsukayama, Berstein, and Ericsson (2011) compared participants accumulated solitary spelling practice to their placement in the 2006 Scripps National Spelling Bee. Two effects compared estimates of accumulated solitary bowling practice against participants’ bowling averages (Harris, 2008; Maynard et al., 2014), and, similarly, one effect from Duffy et al. (2004) compared participants’ solitary darts practice to their single-dart averages. Three effect sizes compared accumulated music practice to group membership based on assessments of skill, drawn from two samples reported by Ericsson et al. (1993) and from one sample reported by Ruthsatz et al. (2008). Finally, one effect from Schultetus and Charness (1997) compared accumulated coached fencing practice to fencing ratings, and one effect from Tuffiash, Roring, and Ericsson (2007) compared estimates of solitary SCRABBLE practice between elite and novice players. The meta-analyses conducted by Macnamara et al. (2014) included a few cases where multiple effect sizes drawn from the same sample required correction for dependent effects. In our selection of effect sizes, each was drawn from an independent sample, so we analyzed the effect sizes as reported.

## Moderator Coding

We now describe coding of the moderator variables denoting the effects including relative versus objective measures of performance and the effects of deliberate versus purposeful practice. For coding the type of performance, we considered performance to be “objective” (*k* = 9; Charness et al., 2005, both effect sizes; de Bruin et al., 2007; Duckworth et al., 2011; Duffy et al., 2004; Gobet & Campitelli, 2007; Harris, 2008; Maynard et al., 2014; and Schultetus & Charness, 1997) if the reported measure was based on a numerical value that represents differences in skill level that can be compared across samples and is widely accepted as an indicator of domain-relevant skill. Performance measures were coded as being “relative” (*k* = 5; Baker et al, 2003; Ericsson et al., 1993, both effect sizes; Ruthsatz et al., 2008; Tuffiash et al, 2007) if they were based on group comparisons between two levels of performers that are widely accepted as having large differences in skill within the same domain, as measured by performance on standardized domain-relevant measures. For coding purposeful versus deliberate practice, we looked for explicit mentions within the original study methods of individualized sessions with coaches or teachers as being included as part of the estimate of solitary practice. If a study did describe individualized instruction sessions as being part of the practice estimate it was considered to be deliberate practice (*k* = 8; Baker et al., 2003; Charness et al., 2005, both effect sizes; Ericsson et al, 1993, both effect sizes; Gobet & Campitelli, 2007; Schultetus & Charness, 1997; Ruthsatz et al., 2008); otherwise, it was classified as purposeful practice (*k* = 6; Duckworth et al., 2011; Duffy et al., 2004; Harris, 2008; Maynard et al., 2014; and Tuffiash et al., 2007).

## Primary Meta-Analysis: The Effects of Deliberate or Purposeful Practice on Performance

All meta-analyses were conducted using the Comprehensive Meta Analysis software (Version 3.3; Biostat, Englewood, NJ) to compute the random-effects weighted average of the selected effects. For our primary analysis, results indicated a significant positive relationship between accumulated purposeful or deliberate practice and performance (*r* = .54, 95% C.I. = [.44, .63], *p* < .001). This suggested that deliberate or purposeful practice accounted for approximately 29% of the variance in performance. We then corrected the estimate from this analysis for attenuation due to imperfect reliability of the performance and practice measures. We followed the same procedure reported by Macnamara et al. (2014), who cited the following formula recommended by Schmidt & Hunter (1996, 1999): corrected *r* = *r_xy_*/(*r_xx_r_yy_*)^1/2^, where *r_xx_* and *r_yy_* are reliability coefficients for x and y. Applying this correction with the estimated reliability of the performance measure set to 0.8 and the estimated reliability of the practice measure set to 0.6 produced a corrected estimated average correlation of *r* = .78, indicating that deliberate or purposeful practice accounted for approximately 61% of the variance in performance.

## Moderator Analysis 1: The Effects of Deliberate or Purposeful Practice on Objective and Relative Measures of Performance

The first moderator analysis investigated whether the method by which skill differences were determined (i.e, relative comparisons of different skill groups or objective measurements of differences in performance) produced significant differences in the estimated relationship between practice and performance. Results indicated both types of performance were significantly correlated with practice (*r_objective_* = .49, *r_relative_* = .65, *p*s < .001), and there was no significant difference between the two correlations (*Q*(1) = 1.45, *p* = .23).

## Moderator Analysis 2: The Differential Effects of Deliberate and Purposeful Practice on Performance

The second moderator analysis investigated whether there were significant differences between average effect size estimates calculated using accumulated amount of deliberate practice and estimates calculated using accumulated amount of purposeful practice. This analysis indicated that practice was positively associated with performance whether it was conducted under the guidance of a coach or teacher (*r_deliberate_* = .56, *p* < .001) or not (*r_purposeful_* = .51, *p* < .001). The difference between these correlations was not statistically significant (*Q*(1) = 0.22, *p* = .64).

## Additional Analyses for Purposeful or Deliberate and Naïve Practice

For the additional meta-analyses examining differences between purposeful or deliberate practice and naïve practice on performance, we looked for additional reported estimates of practice within the previously selected studies that included activities consistent with the definition of naïve practice described in the manuscript. Namely, naïve practice activities included domain-relevant activities motivated by other factors than the goal of improving a particular aspect of performance. We identified eight studies that provided sufficient information to calculate separate effect size estimates for deliberate or purposeful practice and naïve practice, and Table S1 below lists the selected studies and the relevant practice measures. Results indicated that purposeful or deliberate practice may be more strongly correlated with performance (*r* = .51, *p* < .001), as compared to naïve practice (*r* = .39, *p* < .001), but the fact these practice estimates were drawn from the same subjects precluded conducting formal statistical comparisons.

# Procedure for Analysis of Musical Accomplishment in Twins

We reanalyzed the twin sample from the National Merit Twin Study (NMTS), obtaining data from the Henry A. Murray Research Archive at Harvard University ([www.murray.harvard.edu/](http://www.murray.harvard.edu/)). Hambrick and Tucker-Drob (2014) conducted univariate ACE modelling to show that genetic variation significantly predicted musical accomplishment, suggesting substantial heritability and explaining approximately 26% of the variance. Musical accomplishment was defined a dichotomous variable that included at least one of the following: receiving a rating of “good” or better at a school, city, regional, or national music competition, having composed music that had been given at least one public performance, performing with a professional orchestra, or publicly conducting a band or orchestra. Dichotomizing musical accomplishment is problematic, in that it effectively equates the skill level necessary to receive a “good” rating from a school competition with the skill-level necessary to receive the same rating at a national competition. Given that the musicians studied by Ericsson et al. (1993) were enrolled in an elite music academy and had attained a performance level that would allow them to pursue a career as professional musicians, we restricted our definition of musical accomplishment within the NMTS sample to those individuals meeting a similar standard, namely those who had received a rating of good or better at a national competition. Replicating the univariate ACE model with this new definition of musical accomplishment revealed a non-significant genetic effect (*A* = 0.72, *S.E.* = 0.37, *p* > .05).

References

Baker, J., Bagats, S., Büsch, D., Strauss, B., & Schorer, J. (2012). Training differences and selection in a talent identification system. *Talent Development and Excellence, 4*(1), 23-32.

Baker, J., Côté, J., & Abernathy, B. (2003). Learning from the experts: Practice activities of expert decision-makers in sport. *Research Quarterly for Exercise and Sport, 74*, 342-347. doi:10.1080/02701367.2003.10609101

Berry, J., Abernethy, B., & Côté, J. (2008). The contribution of structured activity and deliberate play to the development of expert perceptual and decision-making skill. *Journal of Sports and Exercise Psychology, 30*, 685-708.

Cathey, R. M. (2010). Retrospective practice histories of expert and novice baseball pitchers (Doctoral dissertation). University of South Carolina, Columbia. Available from ProQuest Dissertations and Theses database. (UMI No. 3413286)

Charness, N., Tuffiash, M., Krampe, R., Reingold, E., & Vasyukova, E. (2005). The role of deliberate practice in chess expertise. *Applied Cognitive Psychology, 19*, 151–165. doi:10.1002/acp.1106

de Bruin, A. B. H., Rikers, R. M. J. P., & Schmidt, H. G. (2007). The influence of achievement motivation and chess-specific motivation on deliberate practice. *Journal of Sport & Exercise Psychology, 29*, 561–583.

Duffy, L. J., Baluch, B., & Ericsson, K. A. (2004). Dart performance as a function of facets of practice amongst professional and amateur men and women players. *International Journal of Sport Psychology, 35*, 232–245.

Duckworth, A. L., Kirby, T. A., Tsukayama, E., Berstein, H., & Ericsson, K. A. (2011). Deliberate practice spells success: Why grittier competitors triumph at the national spelling bee. *Social Psychological & Personality Science, 2*, 174–181. doi:10.1177/1948550610385872

Ericsson, K. A., Krampe, R. T., & Tesch-Römer, C. (1993). The role of deliberate practice in the acquisition of expert performance. *Psychological Review, 100*, 363–406. doi:10.1037/0033-295X.100.3.363

Ericsson, K. A. (2018) The Differential influence of experience, Practice, and deliberate practice on the development of superior individual performance of experts In K. A. Ericsson, R. R. Hoffman, A, Kozbelt, & A. M. Williams (Eds.) .2nd Revised Edition of Cambridge handbook of expertise and expert performance (pp. 745-769). Cambridge, UK: Cambridge University Press.

Gobet, F., & Campitelli, G. (2007). The role of domain-specific practice, handedness, and starting age in chess. *Developmental Psychology, 43*, 159–172. doi:10.1037/00121649.43.1.159

Hambrick, D. Z., & Tucker-Drob, E. M. (2015). The genetics of music accomplishment: evidence for gene–environment correlation and interaction. *Psychonomic Bulletin & Review, 22*, 112–120. doi:10.3758/s13423-014-0671-9

Harris, K. R. (2008). Deliberate practice, mental representations, and skilled performance in bowling (Doctoral dissertation). Florida State University. Available from Electronic Theses, Treatises and Dissertations, Diginole Commons. (Paper No. 4245)

Helsen, W. F., Starkes, J. L., & Hodges, N. J. (1998). Team sports and the theory of deliberate practice. Journal of Sport & Exercise Psychology, 20, 12–34. Hendry, D. T. (2012). The role of developmental activities on self determined motivation, passion and skill in youth soccer players (Master’s thesis). The University of British Columbia. Retrieved from <http://hdl.handle.net/2429/43553>

Hendry, D. T. (2012). The role of developmental activities on self determined motivation, passion and skill in youth soccer players (Master’s thesis). The University of British Columbia. Retrieved from http://hdl.handle.net/2429/43553

Hodges, N. J., & Starkes, J. L. (1996). Wrestling with the nature of expertise: A sport-specific test of Ericsson, Krampe, and Tesh-Römer’s (1993) theory of deliberate practice. *International Journal of Sport Psychology, 27*, 400–424.

Howard, R. W. (2012). Longitudinal effects of different types of practice on the development of chess expertise. Applied Cognitive Psychology, 26, 359–369. doi:10.1002/acp.1834.

Hutchinson, C. U., Sachs-Ericsson, N. J., & Ericsson, K. A. (2013). Generalizable aspects of the development of expertise in ballet across countries and cultures: A perspective from the expert-performance approach*. High Ability Studies, 24,* 21-47. [doi: 10.1080/13598139.2013.780966](https://doi.org/10.1080/13598139.2013.780966)

Kopiez, R. & Lee, J. I. (2008). Towards a general model of skills involved in sight reading music. *Music Education Research, 10*, 41–62. doi:10.1080/14613800701871363

Krampe, R. T., & Ericsson, K. A. (1996). Maintaining excellence: Deliberate practice and elite performance in young and older pianists. *Journal of Experimental Psychology: General, 25*, 331–359. doi:10.1037/0096-3445.125.4.331

Law, M. P., Côté, J., & Ericsson, K. A. (2007). Characteristics of expert development in rhythmic gymnastics: A retrospective study. *International Journal of Sport and Exercise Psychology*, *5*(1), 82-103. doi: 10.1080/1612197X.2008.9671814

Lehmann, A. C., & Ericsson, K. A. (1996). Performance without preparation: Structure and acquisition of expert sight-reading and accompanying performance. *Psychomusicology, 15*, 1–29. doi:10.1037/h0094082

Macnamara, B. N., Hambrick, D. Z., & Oswald, F. L. (2014). Deliberate practice and performance in music, games, sports, education, and professions: A meta-analysis. *Psychological Science, 25,* 1608–1618. DOI: 10.1177/0956797614535810

Maynard, D., Hambrick, D. Z., & Meinz, E. J. (2014). Practice vs. play as predictors of individual differences in bowling skill. Unpublished data.

McPherson, G. E. (2005). From child to musician: Skill development during the beginning stages of learning an instrument. *Psychology of Music, 33*(1), 5-35.

Meinz, E. J. (2000). Experience-based attenuation of age-related differences in music cognition tasks. *Psychology and Aging, 15*, 297–312. doi:10.1037/0882-7974.15.2.297

Meinz, E. J., & Hambrick, D. Z. (2010). Deliberate practice is necessary but not sufficient to explain individual differences in piano sight-reading skill: The role of working memory capacity. *Psychological Science, 21*, 914–919. doi:10.1177/0956797610373933

Memmert, D., Baker, J., & Bertsch, C. (2010). Play and practice in the development of sport-specific creativity in team ball sports. *High Ability Studies, 21*, 3–18. doi:10.1080/13598139.2010.488083

Plant, E. A., Ericsson, K. A., Hill, L., & Asberg, K. (2005). Why study time does not predict grade point average across college students: Implications of deliberate practice for academic performance. *Contemporary Educational Psychology, 30*, 96–116. doi:10.1016/j.cedpsych.2004.06.001

Ruthsatsz, J., Detterman, D., Griscom, W. S., & Cirullo, B. A. (2008). Becoming an expert in the musical domain: It takes more than just practice. *Intelligence, 36*, 330-338. doi:10.1016/j.intell.2007.08.003

Schmidt, F. L., & Hunter, J. E. (1996). Measurement error in psychological research: Lessons from 26 research scenarios. *Psychological Methods, 1*, 199-223.

Schmidt, F. L., & Hunter, J. E. (1999). Theory testing and measurement error. *Intelligence*, *27*(3), 183-198.

Schultetus, S., & Charness, N. (1997). Fencing data. Unpublished data. Cited in Deakin, J. M., & Cobley, S. (2003). A search for deliberate practice: Expert performance in sports: Advances in research on sport expertise. In J. L. Starkes & K. A. Ericsson (Eds*.), Expert performance in sports: Advances in research on sport expertise* (pp. 115–136). Champaign, IL: Human Kinetics.

Sonnentag, S., & Kleine, B. M. (2000). Deliberate practice at work: A study with insurance agents. *Journal of Occupational and Organizational Psychology, 73,* 87-102.

Tuffiash, M. (2002). Predicting individual differences in piano sight-reading skill: Practice, performance, and instruction (Unpublished master’s thesis). The Florida State University, Tallahassee.

Ureña, C. A. (2004). Skill acquisition in ballet Dancers: The relationship between deliberate practice and expertise (Unpublished doctoral dissertation). The Florida State University, Tallahassee.
